# *N*-Glycoprofiling Analysis for Carbohydrate Composition and Site-Occupancy Determination in a Poly-Glycosylated Protein: Human Thyrotropin of Different Origins

**DOI:** 10.3390/ijms18020131

**Published:** 2017-02-03

**Authors:** Maria Teresa C. P. Ribela, Renata Damiani, Felipe D. Silva, Eliana R. Lima, João E. Oliveira, Cibele N. Peroni, Peter A. Torjesen, Carlos R. Soares, Paolo Bartolini

**Affiliations:** 1 Biotechnology Center, Instituto de Pesquisas Energéticas e Nucleares, IPEN-CNEN/SP—Avenida Prof. Lineu Prestes, 2242—Cidade Universitária, 05508-000 São Paulo, Brazil; mtribela@ipen.br (M.T.C.P.R.); renata.damiani@biosintesis.com.br (R.D.); felipedouglasichigo@gmail.com (F.D.S.); lima-eliana@hotmail.com (E.R.L.); jeolivei@ipen.br (J.E.O.); cnperoni@ipen.br (C.N.P.); crsoares@ipen.br (C.R.S.); 2 Hormone Laboratory, Oslo University Hospital, 0424 Oslo, Norway; pabusdal@online.no

**Keywords:** *N*-glycoprofiling, *N*-glycans, human thyrotropin (hTSH), carbohydrate site-occupancy, MALDI-TOF-MS, pharmacokinetics

## Abstract

Human thyrotropin (hTSH) is a glycoprotein with three potential glycosylation sites: two in the α-subunit and one in the β-subunit. These sites are not always occupied and occupancy is frequently neglected in glycoprotein characterization, even though it is related to folding, trafficking, initiation of inflammation and host defense, as well as congenital disorders of glycosylation (CDG). For the first time *N*-glycoprofiling analysis was applied to the site-occupancy determination of two native pituitary hTSH, in comparison with three recombinant preparations of hTSH, a widely used biopharmaceutical. A single methodology provided the: (i) average *N*-glycan mass; (ii) mass fraction of each monosaccharide and of sulfate; and (iii) percent carbohydrate. The results indicate that the occupancy (65%–87%) and carbohydrate mass (12%–19%) can be up to 34%–57% higher in recombinant hormones. The average glycan mass is 24% lower in pituitary hTSH and contains ~3-fold fewer moles of galactose (*p <* 0.005) and sialic acid (*p <* 0.01). One of the two native preparations, which had the smallest glycan mass together with the lowest occupancy and GalNAc, sulfate, Gal and sialic acid contents, also presented the lowest in vivo bioactivity and circulatory half-life. The methodology described, comparing a recombinant biopharmaceutical to its native equivalent, can be applied to any physiologically or clinical relevant glycoprotein.

## 1. Introduction

Human thyroid stimulating hormone (hTSH) is a heterodimeric glycoprotein secreted by the pituitary gland, consisting of two non-covalently bound subunits, α and β, in which the carbohydrate portion represents 10%–20% of the total weight. Both subunits can be glycosylated: in the α-subunit at two glycosylation sites (Asn-52 and Asn-78) and in the β-subunit at one glycosylation site (Asn-23) [1,2].

The *N*-linked oligosaccharides (*N*-glycans) of the β-subunit of hTSH, and of glycohormones in general, play a pronounced role in secretion, disulfide bond pairing and the metabolic clearance rate, which is a major factor in determining in vivo bioactivity [3,4]. At the same time, the α-subunit oligosaccharides are essential for signal transduction and participate in β-subunit folding and heterodimer stabilization [1,5,6,7,8]. These *N*-glycans, from a variety of species, differ in branching and, especially, in two terminal modifications: sialylation and sulfation. Such modifications greatly influence the half-life of glycohormones since sialylation protects them against clearance, while sulfation leads to a more rapid clearance rate upon binding to a receptor on the surface of liver endothelial cells [9]. Besides altering the pharmacokinetics of a given hormone, the carbohydrate moiety can also affect its receptor interactions. Human α-subunit Asn^52^ oligosaccharide, located close to the putative receptor-binding region, seems in fact to exert a hormone-specific functional role on the hormone-receptor interaction and receptor activation [10].

The specificity of the hormone action is mostly determined by the β-subunit, but it is also known that determinants in the heterodimer account for the synthesis of hormone-specific carbohydrate structures. The diversity of the oligosaccharide structures that are finally synthesized indicates that the assembly of the α-subunit with the hormone-specific β-subunit, occurring in the endoplasmic reticulum, elicits a hormone-specific processing of *N*-linked oligosaccharides [8,9,11].

Chinese hamster ovary (CHO) cell-derived hTSH is pharmacologically very important, especially for thyroid cancer management, both in the diagnostic follow-up of differentiated thyroid carcinoma and in post-surgical thyroid remnant ablation with radioiodine-131, besides being widely used for in vitro diagnostic applications [12]. This biopharmaceutical is also used to evaluate thyroid reserve capacity and to enhance radioiodine uptake in patients with multinodular goiter [1,13,14,15].

In hTSH as in other glycoproteins, glycosylation sites are not always fully occupied and, during product characterization, the level of occupancy is frequently neglected, even though it has been related to folding, trafficking, initiation of inflammation and host defense, as well as to disease states such as congenital disorders of glycosylation (CDG). This is a family of rare, inherited metabolic syndromes affecting the synthesis, transfer or processing of glycans that can cause motor and intellectual disability and variable multisystemic symptoms. CDG type-I presents impaired early glycosylation steps and, consequently, unoccupied glycosylation sites, while in CDG type-II the defects are more related to glycan processing [16,17,18,19,20,21]. In this context, one cannot overlook the complexity of glycan composition and the challenging task of the simultaneous accurate determination of: (i) protein concentration; (ii) oligosaccharide structures (i.e., the variation of the glycoform profile); and (iii) the glycosylation site occupancy of glycoproteins in general [19,20,22,23,24].

In previous work, *N*-glycoprofiling analysis, based on a novel validated approach, was employed to determine the monosaccharide composition of each glycan, the average glycan mass, the whole glycoprotein mass and the percent carbohydrate moiety [25]. The glycoprotein analyzed, glycosylated human prolactin (G-hPRL), was a very simple model for a mono-glycosylated protein with a unique potential glycosylation site. After separation from the non-glycosylated form (NG-hPRL), it therefore presented 100% occupancy. In the present work, we extend the same approach to a poly-glycosylated protein (hTSH) with three potential glycosylation sites. Because of the parallel application of an accurate molecular mass determination of the glycoprotein via MALDI-TOF-MS, the glycosylation site-occupancy can also be determined in addition to all of the other parameters mentioned above. As in the case of G-hPRL, a thorough comparison between native pituitary and CHO-derived *N*-glycan composition was also carried out, extending the study to five different hTSH preparations: two pituitary and three of recombinant origin. We believe that this type of comparison is extremely important when characterizing a widely used recombinant biopharmaceutical.

## 2. Results

All different *N*-glycan structures (*n =* 64) determined via *N*-glycoprofiling of the five hTSH preparations are reported in Table 1, following the same nomenclature utilized in previous work [25]. Considering the number of structures, the two native preparations (P1, P2) displayed 38–40 different *N*-glycans and the recombinant “humanized” hTSH (R3) displayed 20; the two classical recombinant preparations (R1 and R2) presented exactly the same 15 structures, all of which had similar intensities. In the lower range of glycan molecular masses, only P1 showed a relevant presence of high mannose structures, Man_5–7_GlcNAc_2_ (17.5%), while humanized hTSH was the sole preparation showing a large fraction (37.7%) of high molecular mass tri- and tetra-antennary glycans. It is also noteworthy that N2G2S2 was the most frequent structure, present in all preparations (10%–32%) except in humanized hTSH, while the CHO-derived glycan with the highest intensity was by far N2G2S1 (21%–36%).

**Figure 1:**
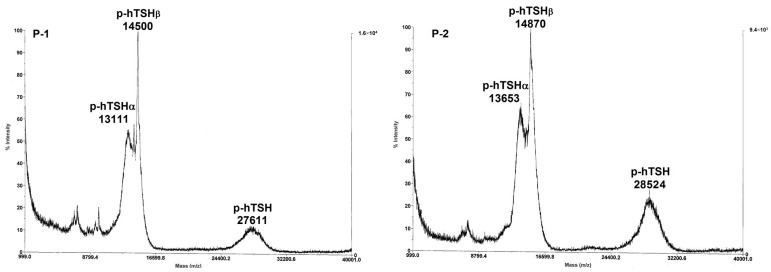

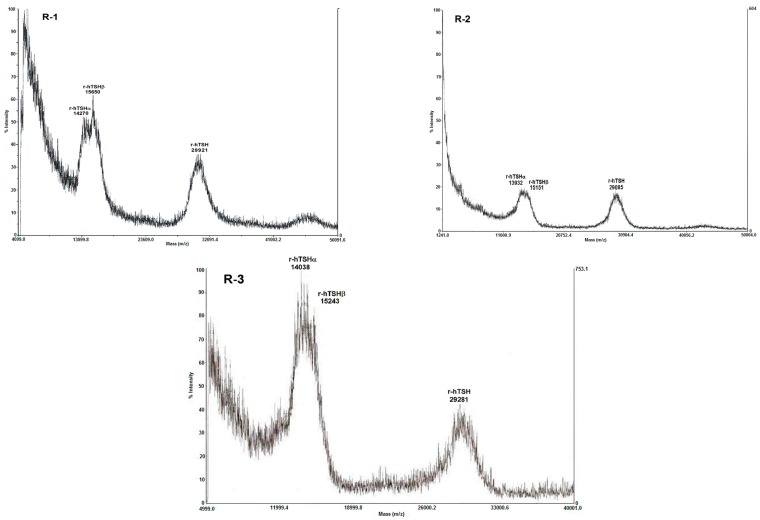
MALDI-TOF mass spectra of native, pituitary (P1, P2) and of recombinant (R1, R2, R3) hTSH preparations.

In Table 2, the following parameters are reported for each hTSH preparation: (i) the glycoprotein molecular mass determined via MALDI-TOF-MS (Figure 1); (ii) the average glycan mass (AGM); (iii) moles of each monosaccharide (and sulfate) per mole of hTSH; (iv) percent of carbohydrate moiety; and (v) occupancy. From these data, average molecular masses of 28,067 ± 645 and 29,429 ± 437 (difference = 4.8%, *p* = 0.067) and average glycan masses of 1800.9 ± 184 and 2227.3 ± 164 (difference = 23.7%, *p* = 0.077), were calculated for the two native and three recombinant hTSH preparations. The number of moles of fucose per mole of hTSH was found to be 2.5-fold higher in the pituitary preparations: 0.74 ± 0.04 versus 0.30 ± 0. On the other hand, the numbers of moles of galactose and sialic acid (SA) per mole of hTSH were found to be about three-fold higher in the recombinant preparations: 5.09 ± 0.48 versus 1.68 ± 0.42 (*p <* 0.005) in the case of galactose and 3.38 ± 0.33 versus 1.18 ± 0.32 (*p <* 0.01) in the case of SA. The average SA/Gal molar ratio showed a low variation and no evident difference between pituitary and recombinant preparations: *X* = 0.68 ± 0.022 (*n =* 5; coefficient of variation (*CV*) = 3.2%). GalNAc and sulfate, only present in P1 and P2, were, respectively, four-fold and seven-fold higher in the latter: as is known, GalNAc-transferases and sulfotransferases are not present in CHO.

The average carbohydrate moiety and occupancy were also higher (37% and 12%, respectively) in the recombinant preparations, though without statistical significance. In the case of the percent carbohydrate moiety in the total glycoprotein molecular weight, it is important to note the good agreement between the present values and those previously determined via carbohydrate composition analysis, a comparison that contributes to validate the proposed methodology.

The biological activities of each hTSH preparation, as determined in the classical in vivo bioassay, are reported in Table 3. The average bioactivity of recombinant preparations was 38% higher, though non-statistically significant (*p* = 0.087). Undoubtedly, one native preparation (P1) showed a statistically significant lower potency compared to the other preparations, either pituitary or recombinant. Interestingly, this same preparation is the one that also presented, by far, the lowest sialylation level.

**Figure 2:**
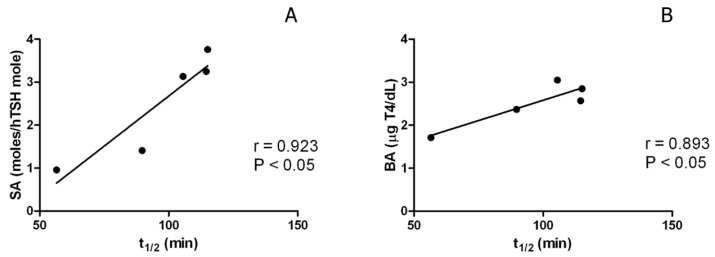
Correlation curves comparing: (**A**) t_1/2_ with sialylation (SA), *Y* = 0.047*X* − 1.98; *r* = 0.923; *p <* 0.05; and (**B**) t_1/2_ with bioactivity (BA), *Y* = 0.019*X* + 0.69; *r* = 0.893; *p <* 0.05, considering all five hTSH preparations.

The two main pharmacokinetic parameters (t_1/2_ and AUC) are reported in Table 4, the average t_1/2_ of the three recombinant hormones being 25% higher than that of the pituitary preparations, a statistically significant difference (*p <* 0.02). We can observe, moreover, in Figure 2, the significant correlation existing between t_1/2_ and sialylation (*p <* 0.05) and between t_1/2_ and bioactivity (*p <* 0.05) for the five preparations. Noteworthy are the remarkably lower coefficients of variation related to the average bioactivity and half-life of the three recombinant preparations (Table 3 and Table 4), showing the possibility of attaining a better controlled quality in the case of CHO-derived hTSH.

## 3. Discussion

Thanks to the methodology based on *N*-glycoprofiling structural analysis previously applied to mono-glycosylated G-hPRL [25], it has been possible, with the addition of MALDI-TOF-MS total molecular mass determination, to apply the same procedure to the poly-glycosylated protein hTSH. The quantitative monosaccharide determination for all preparations and the corresponding molar ratios, all derived from *N*-glycoprofiling analysis, were based on a stoichiometric calculation which considered the molecular mass and relative quantification (percent intensity) of each detected *N*-glycan, as previously reported [17,25]. Five different hTSH preparations, two of pituitary origin, two from conventionally modified CHO cells and one from partially humanized CHO cells, could thus be evaluated and compared.

Carbohydrate compositional analysis comparing pituitary and recombinant hTSH had alrerady been carried out by Szkudlinski et al. [26] and by Cole et al. [27] without, however, defining glycan structures. The first detailed structural characterization of N-linked oligosaccharides, only from recombinant hTSH, was reported by Morelle et al. [28], who identified 12 different *N*-glycans and also described their microheterogeneity at each glycosylation site. In previous work, our research group reported *N*-glycoprofilings of recombinant human thyrotropin obtained under different culture conditions, identifying 13–20 different structures [7,29] and of humanized recombinant hTSH (hlsr-hTSH), identifying 20 structures [30]. The comparison between the *N*-glycoprofilings of a native glycoprotein and of its equivalent recombinant form was carried out by our group only for G-hPRL. The present work is therefore the first to compare native with recombinant (CHO-derived) hTSH preparations directly, determining the site occupancy and all mentioned carbohydrate compositional parameters, together with bioactivity and pharmacokinetics.

Our calculations were based on several literature reports that confirm the stability and reproducibility of the amino acid structure (protein “backbone”) versus the great variability existing in the glycosylation pattern; indeed, some authors clearly exclude any major importance of amino acid sequence heterogeneity [26,27,28]. A direct proof of protein moiety stability comes from Cole’s calculation of Thyrogen^®^ molecular mass, providing a value of 29,660 Da [27], obtained by adding the experimentally determined carbohydrate structures to the theoretical backbones of the α- and β-peptides. Twenty years later and in a completely different context, we obtained a mass value of 29,921 Da for Thyrogen^®^ via MALDI-TOF-MS, which is only 0.88% higher than the molecular mass first calculated by Cole et al. for the same biopharmaceutical.

The data for the two classical recombinant hTSH preparations indicate that their comparative glycan panel is highly homogeneous. R1 and R2 both present the same 15 structures, the intensities of which show a highly significant correlation (*r* = 0.9822; *p <* 0.001; for a degree of freedom, *DF* = 13). Considering that these two recombinant preparations were synthesized in completely different laboratories (Genzyme, Framingham, MA, USA and IPEN-CNEN, São Paulo, Brazil), with totally different cultivation and purification processes (industrial versus laboratorial), and that the *N*-glycoprofiling analyses were carried out on an inter-assay basis, we can conclude that the *N*-glycan structures and their relative abundance are mostly, if not entirely, based on the protein amino acid sequence and host-cell type. We reached similar conclusions in previous work, analyzing the high similarity of the isoelectric focusing profiles of pituitary and recombinant hTSH preparations, as well as of different origins [7,8]. In R1 and R2, the two most abundant structures are N2G2S1 and N2G2S2 (26.3%–36.1%), perfectly matching the two most abundant structures found by Morelle et al. [28] in their r-hTSH preparation, which was also CHO-derived and from a third manufacturer. It is therefore evident that recombinant glycoproteins can be obtained under more controlled and reproducible conditions.

The 3rd recombinant preparation studied here (hlsr-hTSH) is quite different, since it is derived from a CHO strain modified by the introduction of an α2,6-sialyltransferase. This enzyme, which is present in human thyrotropes but not in CHO cells, introduces α2-6 linkages between SA and galactose, yielding in this way a form of “humanized” recombinant hTSH. This preparation confirmed N2G2S1 as the most abundant structure (21.4%) and also had 42.9% of tri-antennary and tetra-antennary structures, which were present only in much smaller amounts (2.7%–20.9%) in the other four preparations.

In the two pituitary preparations, we found a much higher heterogeneity than in R1 and R2, although the number of *N*-glycan structures was quite similar: 40 and 38 for P1 and P2, respectively. The only feature clearly in common with the two classical recombinant preparations was the richest presence of N2G2S2: 10% and 14.1% for P1 and P2, respectively. Interestingly, P1, P2 and R3 shared practically the same percent of fucosylated glycans (36, 35.2 and 37.9), while R1 and R2 presented much smaller amounts of these residues (11.9% and 13.5%). This confirms the data of Capone et al. [25] for higher G-hPRL fucosylation in native in comparison with recombinant hormone, though both levels were much higher than in hTSH: 90.5% and 75.4%, respectively.

As reported in the literature [1,28,31], recombinant hTSH is in general much more sialylated than the native hormone. This terminal modification is positively correlated with t_1/2_ and, consequently, with in vivo bioactivity. This was confirmed in Figure 2 and in our analysis of P1 and P2 (with 41.2% and 50.8% of sialylated structures) versus R1, R2, and R3 (with 94.2%, 90.9% and 86.3%) and by the corresponding number of moles of sialic acid per mole of hTSH shown in Table 2. It is noteworthy that, in hypothyroidism, plasma TSH has been shown to contain less core fucose [32] and/or more highly sialylated glycans [33], which can be viewed as adaptive responses to the degree of the disease. These alterations, influencing hormone action, should be considered when synthesizing or controlling biopharmaceuticals intended for repeated parenteral administration.

Sulfated glycans, which are only present in native glycoproteins, occurred in a much greater amount in P2 than in P1 (55.7% vs. 13.7%, respectively), a four-fold higher difference that becomes six-fold higher if one considers the number of moles of sulfate per mole of hTSH. Due to the laborious purification process that is necessary for obtaining hTSH from human pituitaries, it has been suggested that terminal modifications, such as sulfate and sialic acid, can be partially lost during this procedure. Alternatively, lability and post-ionization loss could occur during mass spectrometry [34]. The dramatic difference observed in the case of G-hPRL, in which only 1.7% of *N*-glycans were found to be sialylated in the native versus 80.5% in the recombinant preparation, seems to favor this hypothesis. This does not appear to apply to the present case, at least for sialic acid, since the previously reported range of values for the SA/Gal molar ratio of hTSH (0.60–0.68) [7,27,30] agrees with the present average value (SA/Gal = 0.68 ± 0.022; *CV* = 3.2%; *n* = 5), with no indication of bias between the pituitary and recombinant preparations. Sialylation is indeed much lower in pituitary than in recombinant glycoproteins, mainly because of the lower level of galactose in the native material. The SA/Gal ratio, a parameter that was introduced for Thyrogen^®^ (Genzyme) characterization, is therefore essentially identical in the two forms of hTSH and should be greatly altered in the case of a dramatic de-sialylation [27].

The in vivo bioassay scheme set up in previous work [30,35] based on optimized single dose injections in 10 mice per preparation was applied here and provided a good precision (*CV* = 0.8%–27%), especially considering the in vivo variability. A direct, absolute experimental parameter (circulating T4 levels expressed in µg/dL) was privileged in comparison with relative potency calculation based on arbitrary reference preparations. In these bioassay data, it is possible to observe a tendency towards higher activities for the recombinant preparations, although without statistical significance. On the other hand, circulatory half-life, directly related to in vivo bioactivity [26,36], was significantly correlated with this parameter and with sialylation, considering all five preparations: it is worth recalling that the average value of t_1/2_ was significantly higher in the recombinant hormones. Moreover, comparing the two native preparations (P1 and P2) for their bioactivity, t_1/2_, moles of galactose and of sialic acid, we observed constant differences of 39%–59% in favor of P2, which confirms the existence of a correlation between these parameters.

Regarding occupancy, a parameter that can be clinically relevant for glycoproteins, in the case of our hTSH preparations no remarkable difference was found. Whether the existence of a 34% difference in occupancy between, for example, P1 and R1 might have some effect on hormone action is difficult to investigate, considering all the other structural alterations that are also present. It will be important, in a next step, to study the qualitative and quantitative occupancy at each of the three glycosylation sites, since full appreciation of the mode of action of hTSH requires complete knowledge of its structure. Morelle et al. [28] have shown, in fact, that *N*-glycan structures are different at each glycosylation site of the α-subunit, suggesting that a modulation of hTSH bioactivity may reside in the nature of this heterogeneity. This study will be done by either separating the α- and β-subunits prior to *N*-glycoprofiling analysis or by using glycopeptide analysis to complement the analytical work.

In conclusion, all of the data, taken together, point to a higher in vivo bioactivity for recombinant hTSH, justified on the basis of several structural elements. At the same time, surprisingly, one of the two pituitary preparations is approaching some of the characteristics of the recombinant hormones. Again, we must emphasize the higher variation (i.e., lower control and consistency) typical of native preparations. In a more general sense, the successful application of the proposed analytical procedures demonstrates that it is possible to carry out a rapid and efficient evaluation of the structure of a glycoprotein. This is extremely important for quality control and for the potency and safety assessment of recombinant biopharmaceuticals, which can be quite different from native, human biological material. We have in fact compared two native hTSH preparations (very difficult now to obtain with good quality), with different CHO-derived preparations (now a widely used biopharmaceutical). This serves as an alert as to the danger of neglecting composition and biological behavior of the native human hormone and sometimes continuing to administer unnatural molecular structures, no matter whether they are more or less bioactive. At the same time, the methodology and acquired knowledge should be extremely valuable for the future engineering of glycoprotein analogs aimed at developing new diagnostic and therapeutic applications.

## 4. Materials and Methods

### 4.1. hTSH Preparations

The two pituitary preparations of hTSH were obtained from the National Hormone and Pituitary Program (Torrance, CA, USA) and from the Oslo University Hospital (Oslo, Norway). The native hormone was obtained according to classical procedures by removing human pituitary glands at autopsy and storing them in liquid nitrogen until the extraction procedure [37,38,39]. Of the three recombinant preparations, one is a commercial biopharmaceutical (Thyrogen^®^ from Genzyme Corporation, Framingham, MA, USA) [27], while the other two were from our laboratory at IPEN-CNEN/SP (São Paulo, Brazil), prepared and characterized as previously described [29,30,35,36,40]. As mentioned the human-like sialylated recombinant human thyrotropin (hlsr-hTSH) was obtained in a particular CHO cell line, previously modified by the introduction of rat α2,6-sialyltransferase [30]. The purity of all utilized preparations was >98%, all having been lyophilized and stored at −20 °C. The declared Thyrogen^®^ formulation included mannitol, sodium phosphate and sodium chloride. The pituitary preparations were totally pure. The IPEN preparations were lyophilized in 0.15 M NaCl, 0.02 M sodium phosphate buffer, pH 7.0. The origin of each preparation is not declared, being identified only as of pituitary or recombinant origin: P-1, P-2, R-1, R-2, R-3. Part of the *N*-glycoprofiling data referring to R3 have been published [30]; this preparation is therefore identified as hlsr-hTSH.

### 4.2. In Vivo Bioassay

All assays were conducted in accordance with the national protection laws on animal welfare.

The biological activity of the different hTSH preparations was determined by an in vivo bioassay in albino laboratory-bred strain (BALB)/c mice in which TSH-induced T_4_ is measured after a 5-day suppression of endogenous TSH by T_3_ administration. A single dose (10 µg hTSH per mouse, injected intraperitoneally) and 10 mice were utilized for each hTSH preparation. Plasma samples were obtained at 6 h post injection. The T4 concentrations were determined using T4 RIA kits (Siemens, Los Angeles, CA, USA), according to manufacturer’s instructions [41].

### 4.3. Immunoradiometric Assay (IRMA)

Human TSH IRMA was carried out by an in-house methodology, utilizing a secondary hTSH standard calibrated against the international standard of pituitary hTSH (WHO 80/558, 4.93 IU/mg), as described [12,29].

### 4.4. Pharmacokinetic Studies

Single doses of the different hTSH preparations (2 µg/200 µL) were administered to BALB/c mice by intraperitoneal (i.p.) injection (*n =* 5 animals/group). Plasma samples were withdrawn at 30, 60, 90, 120, 180, 240 and 300 min post injection. Human TSH concentrations were determined by IRMA and expressed as the percentage of the maximum concentration [29].

### 4.5. Mass Spectrometry for Molecular Mass Determination

MALDI-TOF-MS molecular mass determination of heterodimeric hTSH was carried out by American International Biotechnology (AIBio Tech. Services, Richmond, VA, USA) using sinapinic acid as the matrix and a Voyager-DE BioSpectrometry Workstation from Applied Biosystems (Foster City, CA, USA).The molecular mass of the heterodimer could be further confirmed by summing the α- and β-subunit mass values, simultaneously detected in the spectrogram, as found in Laidler et al. [42] and in previous work from our group [8,36,43].

### 4.6. N-Glycosylation Profiling by MALDI-TOF-MS

The analysis of the *N*-glycan structures was carried out by Proteodynamics SARL (Clermont-Ferrand, France). Approximately 400–500 µg of each one of the two pituitary and of the three recombinant hTSH preparations mentioned above were used for the analysis (see Supplementary File 1).

#### 4.6.1. Glycosidase Digestion and Permethylation of *N*-Glycans

Glycoproteins were denatured in 0.5% sodium dodecyl sulfate (SDS) and 1% β-mercaptoethanol (90 °C, 10 min) and deglycosylated by enzymatic digestion, treating for 15 h at 37 °C with 20 units of PNGase F from Roche Applied Science (Mannheim, Germany), according to Fogli et al. [17]. Deglycosylation was monitored on a NuPage gradient of 4%–12% MES (Invitrogen, Carlsbad, CA, USA) by Comassie blue staining.

A specifically adapted permethylation protocol based on Yu et al. [44] and previously described [25], was employed to separate sulfated from non-sulfated or phosphorylated *N*-glycans of pituitary hTSH. A 25% acetonitrile fraction contained sulfated glycans, present only in the pituitary products, while non-sulfated and phosphorylated glycans were found in the 50% acetonitrile fraction. Eluted *N*-glycans were lyophilized and submitted to MALDI-TOF-MS analysis.

#### 4.6.2. MALDI-TOF Analysis of *N*-Glycans

Mass spectra of permethylated *N*-glycans resuspended in 20 µL of 50% methanol/water were acquired in the positive reflector mode (*m*/*z* range 1000–5000 Da) on MALDI-TOF DE PRO (ABsiex, Inc., Framingham, MA, USA) with DHB as matrix (10 mg/mL, ratio 1:1). The spectra were obtained by accumulation of 500 shots and were calibrated with an external standard. MALDI-MS data were processed using DataExplorer 4.0 to generate files listing *m*/*z* values and to compare intensities. Interpretation of glycan structures corresponding to monoisotopic masses was performed using ExPaSy GlycoMod (http://web.expasy.org/glycomod/) and GlycoWorkBench. MALDI-PSD fragmentation was performed on selected ions to confirm structures.

A correlation factor between the spectra of sulfated and non-sulfated/phosphorylated glycans was calculated in order to obtain a relative quantification of all *N*-glycans present in the pituitary preparations. This correlation factor was determined in two ways: (i) by adding an internal calibration standard to normalize peak intensities of the two spectra; and (ii) by mixing the two glycan fractions and using the ratio between the heights of the dominant peaks of each fraction. Ten *N*-glycan structures, determined in the two native hTSH preparations, and representing 13.8%–14.3% of the total, contained different combinations of GlcNAc and GalNAc that could not be distinguished. In these cases, the most unusual branches, never or seldom reported in the literature [17,28,45,46,47], were not considered.

#### 4.6.3. Average *N*-Glycan Mass and Monosaccharide Molar Ratio Determination on the Basis of Glycoprofiling

The glycoprofiling and the relative percent intensity of each determined glycan were used to calculate the average *N*-glycan mass that is present in the hTSH molecule, for each different preparation. Through this stoichiometric approach the contribution of each monosaccharide to each glycan can also be calculated. All calculations have been carried out as detailed in a previous work [25].

### 4.7. Site-Occupancy and Mass of the Carbohydrate Moiety Determination

Having determined the Average Glycan Mass (AGM) on the basis of glycoprofiling and the total molecular mass of the glycoprotein by MALDI-TOF-MS, the site-occupancy can be calculated according to the following relation:

AGM × Number of Occupied sites = Mass of Carbohydrate Moiety

where the mass of the carbohydrate moiety in the hTSH molecule can be obtained from the relation:

Mass of Carbohydrate Moiety = Molecular Mass - hTSH protein backbone

The hTSH protein backbone is 24,330 Da (10,777 Da for the α- and 13,553 Da for the β-subunit peptide), as determined by Cole et al. (personal communication), considering the amino acid sequence of r-hTSH (Thyrogen^®^) and of its individual subunits [27]. Knowing the occupancy, it is then possible to determine each monosaccharide (or sulfate)/hTSH molar ratio.

## Figures and Tables

**Table 1:** Different *N*-glycan structures and relative intensities of two native and three recombinant preparations of human thyrotropin**.**

*N*-Glycan ^1^	Underivatized Mass (-H_2_O) (Da)	Relative Intensity of Each *N*-Glycan per Each Preparation (%)				
		P1	P2	R1	R2	R3 ^2^
(1) 0	892.3	2.0				
(2) F1	1038.4	1.9				
(3) M1	1054.4	3.7				
(4) M2	1216.4	7.0				
(5) N1F1	1241.5	2.7				
(6) N1G1	1257.5	1.3				
(7) N2	1298.5	0.4				0.8
(8) N1Gn1(SO4)	1378.4	2.0	5.1			
(9) M3	1378.5	9.0				
(10) M1N1G1/M2N1	1419.5	0.9				
(11) N2F1	1444.5	3.2	0.3			0.7
(12) N2G1	1460.5	1.9	0.4	0.4	1.9	1.4
(13) N1Gn1F1(SO4)	1524.5	0.8	1.5			
(14) N1G1 Gn1(SO4)	1540.5	2.2	5.2			
(15) M4	1540.5	1.5				
(16) N1G1S1	1548.5	8.2	1.8			
(17) N2Gn1(SO4)	1581.5	2.1	6.7			
(18) M2N1G1/N1M3	1581.6	0.8				
(19) N2G1F1	1606.6	2.4	1.0			1.9
(20) N2G2	1622.6	2.1		5.0	6.2	3.8
(21) N3F1	1647.6	2.0	1.8			
(22) M1N1Gn1F1(SO4)	1686.6		0.7			
(23) N1G1S1F1	1694.6	4.1	1.1			
(24) M2N1Gn1(SO4)	1702.5	0.3	2.5			
(25) M1N1G1S1	1710.6			1.2	1.5	
(26) N2Gn1F1(SO4)	1727.6		1.4			
(27) N2G1Gn1(SO4)	1743.6	1.8	3.6			
(28) N2G1S1	1751.6		1.3	0.5	3.1	12.3
(29) N2G2F1	1768.6	0.6	0.6			1.6
(30) N2Gn2(SO4)	1784.6	0.6				
(31) N2G1Gn1F1	1809.7	1.5	0.8			
(32) N2Gn2(SO4)2	1864.6		3.4			
(33) N2G1Gn1F1(SO4)	1889.6		4.6			
(34) N2G1S1F1	1897.7	2.7	1.0			
(35) N2G2S1	1913.7	1.9	0.9	36.1	26.3	21.4
(36) N2Gn2F1(SO4)	1930.7	0.3	1.6			
(37) N2G1Gn1F2	1955.7	1.8	0.8			
(38) N2G1Gn1S1	1970.7	1.6	0.7			
(39) N3G3	1987.7					2.4
(40) N2Gn2F1(SO4)2	2010.6	0.6	4.4			
(41) N3G2Gn1	2028.7		1.3			
(42) N2G1Gn1S1(SO4)	2034.7		14.0			
(43) N2G2S1F1	2059.7	1.3		3.4	4.1	11.0
(44) N2G1Gn1S1F1	2100.7	5.5	4.2			
(45) N3G2F2	2117.8			0.5	1.0	
(46) N3G2Gn1F1	2174.8		1.4			
(47) N2G1Gn1S1F1(SO4)	2180.7		3.5			
(48) N2G2S2	2204.8	10.0	14.1	31.9	31.2	0.6
(49) N2G1Gn1S2	2245.8	2.6	2.3			
(50) N3G3S1	2278.8			2.7	3.7	
(51) N2G2S2F1	2350.8	1.9	2.1	5.3	4.8	1.5
(52) N3G3S1F1	2424.9			0.5	1.1	
(53) N4G4F1	2498.9					1.0
(54) N3G2S2F1	2553.9	1.3	0.5			
(55) N3G3S2	2569.9			6.9	7.7	1.8
(56) N4G3Gn1S1	2685.0		0.8			
(57) N3G3S2F1	2716.0			1.5	1.6	
(58) N4G3Gn1S1F1	2831.0		1.0			
(59) N3G3S3	2861.0		0.6	3.5	4.9	5.0
(60) N4G4S2	2935.0					2.6
(61) N3G3S3F1	3007.1	1.4	0.9	0.7	0.9	9.5
(62) N4G4S3	3226.1					3.2
(63) N4G4S4	3517.2					6.7
(64) N4G4S4F1	3663.3					10.7
**Fucosylated glycans**		**36.0**	**35.2**	**11.9**	**13.5**	**37.9**
**Sialylated**		**41.2**	**50.8**	**94.2**	**90.9**	**86.3**
**Sulfated**		**13.7**	**55.7**	**-**	**-**	**-**
**Bi-antennary**		**46.8**	**73.7**	**82.6**	**77.6**	**57.0**
**Triantennary**		**4.7**	**6.5**	**16.5**	**20.9**	**18.7**
**Tetraantennary**		**-**	**1.8**	**-**	**-**	**24.2**

^1^ Abbreviations for *N*-glycans were made by not considering the basic pentasaccharidic nucleus (“zero”) and adding all other monosaccharides, as stated in Table 1, in the following order: Man (**M**); GlcNAc (**N**); Gal (**G**); GalNAc (**Gn**); NeuAc/sialic acid (**S**); Fuc (**F**). For example, NeuAc_1_ Gal_1_ GlcNAc_2_ Fuc_1_ + Man_3_ GlcNAc_2_, becomes **N2G1S1F1**; ^2^ This *N*-glycoprofiling panel, specifically for hlsr-hTSH, is from Damiani et al. (2009). “-” not present.

**Table 2:** Average *N*-glycan mass, monosaccharide or sulfate/hTSH molar ratio, carbohydrate moiety and occupancy determination based on *N*-glycoprofiling and MALDI-TOF-MS analysis of the different hTSH preparations.

hTSH Preparation	Molecular Mass by MALDI-TOF-MS (Da)	Average Glycan Mass (Da)	Monosaccharide or Sulfate Moles/hTSH Mole							Carbohydrate Moiety (%)		Occupancy Occupied Sites	(%)
			Fuc	GalNAc	GlcNAc	Gal	Man	SA	Sulfate				
P-1	27,611	1670.5	0.71	0.45	6.51	1.39	7.57	0.96	0.23	11.88 ^1^	(11.2) ^2^	1.96/3	65.3
P-2	28,524	1931.4	0.77	1.84	8.59	1.98	6.84	1.41	1.54	14.70 ^1^	(14.3) ^2^	2.17/3	72.3
R-1	29,921	2128.2	0.30	0	11.04	5.63	8.08	3.76	0	18.68 ^1^	(18.0) ^2^	2.63/3 ^3^	87.7
R-2	29,085	2137.6	0.30	0	9.43	4.72	6.85	3.13	0	16.35 ^1^	(16.5) ^2^	2.22/3	74.0
R-3	29,281	2416.2	0.74	0	9.93	4.92	6.67	3.25	0	16.91 ^1^	(16.5) ^2^	2.05/3	68.3

^1^ Determined considering hTSH protein backbone = 24,330 Da; ^2^ Carbohydrate moiety previously determined via compositional analysis; ^3^ 2.27/3 when determined via the Man/hTSH molar ratio.

**Table 3:** T4 levels obtained in in vivo bioassays (*N =* 10 mice) after administration of the different hTSH preparations.

Preparation	T4 Level (µg/dL)
P-1	1.71 ± 0.13
P-2	2.37 ± 0.64
R-1	2.85 ± 0.34
R-2	3.05 ± 0.70
R-3	2.57 ± 0.64

Average bioactivity of the P preparations: *X* = 2.04 ± 0.47 (*CV* = 23%); Average bioactivity of the R preparations: *X* = 2.82 ± 0.24 (*CV* = 8.5%).

**Table 4:** Pharmacokinetic parameters for the different hTSH preparations.

Preparation	No. of Assays	t_1/2_^1^ (min)	Signif. Level	AUC ^2^ (µg∙min/mL)	Signif. ^3^ Level
P-1	2	56.5 ± 15.7	*p <* 0.05	11,463 ± 682.0	*p <* 0.05
P-2	2	89.7 ± 0.42	*p <* 0.05	14,270 ± 2147.1	NS
R-1	3	115.1 ± 3.12	-	16,155 ± 1398.1	-
R-2	3	105.5 ± 18.4	NS	15,555 ± 1197.8	NS
R-3	3	114.5 ± 26.9	NS	16,976 ± 1720.0	NS

Average t_1/2_ for the P preparations: *X* = 73.1 ± 23.4 (*CV* = 32%); Average t_1/2_ for the R preparations: *X* = 111.7 ± 5.38 (*CV* = 4.8%). **^1^** Circulatory half-life*.* ^2^ Area under the curve (AUC). ^3^ The level of significance was calculated versus the preparation with the highest t_1/2_. “-”, reference preparation; NS, non-significant.

## Supplementary Materials

Supplementary materials can be found at www.mdpi.com/1422-0067/18/2/131/s1.
