# Supplementary material for: N-Glycoprofiling Analysis for Carbohydrate Composition and Site-Occupancy Determination in a Poly-Glycosylated Protein: Human Thyrotropin of Different Origins
Source: Int J Mol Sci. 2017 Feb 3;18(2):131. doi: 10.3390/ijms18020131 (PMC5343769; doi:10.3390/ijms18020131)
Supplement: Supplementary file 1 [file ijms-18-00131-s001.pdf]

N-GLYCOPROFILING of a recombinant and a native human TSH sample
(FAPESP Project # 2011:2295-3)

Sample Identification

***Sample 1 (rec-TSH)**: Recombinant hTSH from CHO cells; 456.3 µg lyophilized; composition of buffer before lyophilization: 3.3 mg Na_2HPO_4, 2.5mg NaH_2PO_4, 8 mg NaCl ; 99% purity*
***Sample 2 (pit-TSH)**: Pituitary hTSH; 500 µg lyophilized; 99% purity*

Customer:	*FAPESP*<br>*Project # 2011/2295-3*<br>*Rua Pio XI, 1500*<br>*São Paulo*<br>*Brazil*
Contact person:	*Dr Maria Teresa Ribela*

Instrument : MALDI-TOF Voyager DE PRO AB Sciex

TABLE OF CONTENTS

1. Analysis of recombinant hTSH 3
1.1. Experimental 3
1.1.1. Glycosidase digestion & permethylation of N-glycans 3
1.1.2. MALDI TOF analysis of N-glycans 3
1.2. Results 4
1.2.1. MALDI-TOF MS spectra of permethylated N-glycans 4
1.2.2. Assignment 4
1.2.3. Interpretation 4
1.2.4. Relative intensity (%) 5

2. Analysis of pituitary hTSH 7
2.1. Experimental 7
2.1.1. Glycosidase digestion & permethylation of N-glycans 7
2.1.2. MALDI TOF analysis of N-glycans 7
2.2. Results 8
2.2.1. MALDI-TOF MS spectra of permethylated N-glycans 8
2.2.2. Assignment 9
Assignment of sulphated N-glycans in the 25% acetonitrile fraction (V22686) 9
Assignment of N-glycans in the 50% acetonitrile fraction (V22688) 9
2.2.3. Interpretation 10
Interpretation of sulphated N-glycans in the 25% acetonitrile fraction (V22686) 10
Interpretation of N-glycans in the 50% acetonitrile fraction (V22688) 10
2.2.4. Relative intensity (%) 11
Relative intensity of sulphated N-glycans in the 25% acetonitrile fraction (V22686) 11
Relative intensity of N-glycans in the 50% acetonitrile fraction (V22688) 11
2.2.4. Establishment of a global semi-quantitative profile of N-glycan structures of pit-TSH 12
3. Conclusions 15

1. Analysis of recombinant hTSH

1.1. Experimental

1.1.1. Glycosidase digestion & permethylation of N-glycans

The recombinant TSH sample rec-TSH (456.3 µg) was suspended in 500 µl of 50 mM phosphate buffer pH 7.5 and concentrated by vivaspin 500 (5kDa), denatured in 0.5% sodium dodecyl sulfate (SDS), 1% β mercaptoethanol (90°C, 10 min) and deglycosylated by enzymatic digestion 15 hours with 20 units of PNGase F (PROMEGA V483A) at 37°C in 50 mM phosphate buffer, pH 7.5 (SOP P27/1).
Deglycosylation was controlled on NuPage gradient 4-12% (Invitrogen), MES buffer, with coomassie staining.

N-glycans were purified on Ultra Clean SPE Carbograph (ALLTECH) according to SOP P24/2. After elution with 25% acetonitrile, 0.1%TFA, the *N*-glycans were lyophilised before permethylation. Permethylation using sodium hydroxide, DMSO, ICH3 procedure was performed according to SOP P29/2. After derivatization, the reaction products were purified on C18 Sep Pak Plus (WATERS) and lyophilised before MALDI TOF MS analysis.

1.1.2. MALDI TOF analysis of N-glycans

The purified permethylated glycans were solubilised with 20µl of 50:50 methanol/water. 1µl of each batch was mixed with 2µl of 2,5 DHB (LaserBiolabs) matrix solution (10mg/ml 50:50 methanol/water).
Positive ion reflectron MALDI mass spectra were acquired on a VOYAGER DE PRO (AB Sciex).The spectra were obtained by accumulation of 500 shots and were calibrated with an external standard (CalMix 3, AB Sciex). The acceleration and reflector voltage conditions were set up as follows: target voltage 20kV, first grid 75% of target voltage, Delayed extraction 150ns.
Interpretation of glycan structures corresponding to monoisotopic masses was performed using EXPAZY GlycoMod tool and GlycoWorkBench.

1.2. <u>Results</u>

1.2.1. MALDI-TOF MS spectra of permethylated N-glycans

rec-TSH

V22684 cal 22683

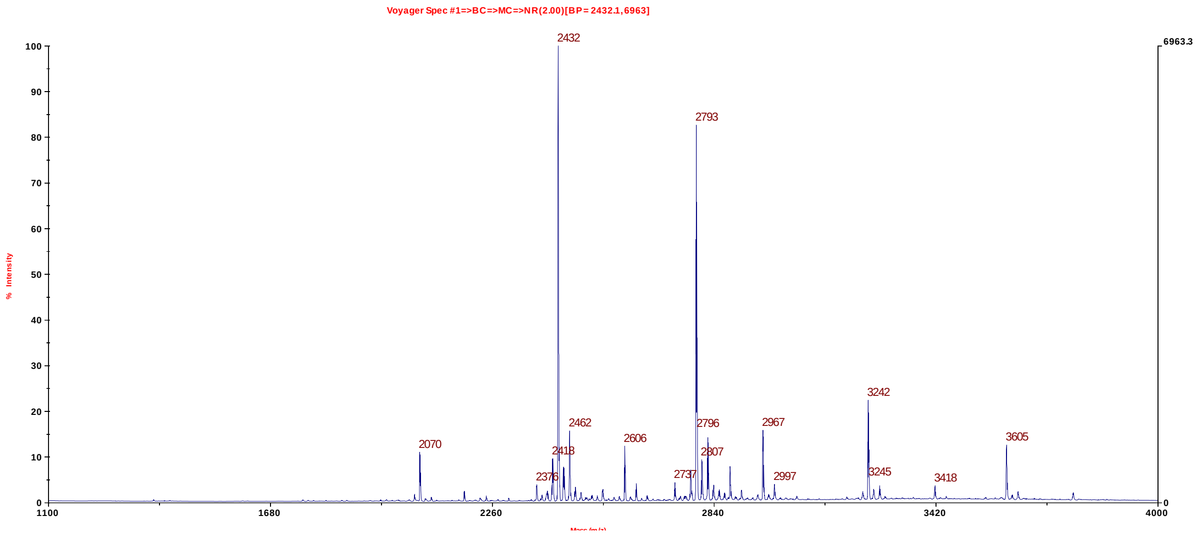

1.2.2. Assignment

Theoretical mass $[M+Na]^+$	rTSH v22684	Assignment
1865,9	1866,0	$(Hex)_1$ $(HexNAc)_2$ + $(Man)_3$ $(GlcNAc)_2$
2070,0	2070,0	$(Hex)_2$ $(HexNAc)_2$ + $(Man)_3$ $(GlcNAc)_2$
2186,1	2186,1	$(Hex)_2$ $(HexNAc)_1$ $(NeuAc)_1$ + $(Man)_3$ $(GlcNAc)_2$
2227,1	2227,1	$(Hex)_1$ $(HexNAc)_2$ $(NeuAc)_1$ + $(Man)_3$ $(GlcNAc)_2$
2431,2	2431,2	$(Hex)_2$ $(HexNAc)_2$ $(NeuAc)_1$ + $(Man)_3$ $(GlcNAc)_2$
2605,3	2605,2	$(Hex)_2$ $(HexNAc)_2$ $(Dehex)_1$ $(NeuAc)_1$ + $(Man)_3$ $(GlcNAc)_2$
2663,3	2663,3	$(Hex)_2$ $(HexNAc)_3$ $(Dehex)_2$ + $(Man)_3$ $(GlcNAc)_2$
2792,4	2792,3	$(Hex)_2$ $(HexNAc)_2$ $(NeuAc)_2$ + $(Man)_3$ $(GlcNAc)_2$
2880,4	2880,3	$(Hex)_3$ $(HexNAc)_3$ $(NeuAc)_1$ + $(Man)_3$ $(GlcNAc)_2$
2966,5	2966,4	$(Hex)_2$ $(HexNAc)_2$ $(Dehex)_1$ $(NeuAc)_2$ + $(Man)_3$ $(GlcNAc)_2$
3054,5	3054,5	$(Hex)_3$ $(HexNAc)_3$ $(Dehex)_1$ $(NeuAc)_1$ + $(Man)_3$ $(GlcNAc)_2$
3241,6	3241,4	$(Hex)_3$ $(HexNAc)_3$ $(NeuAc)_2$ + $(Man)_3$ $(GlcNAc)_2$
3415,7	3415,5	$(Hex)_3$ $(HexNAc)_3$ $(Dehex)_1$ $(NeuAc)_2$ + $(Man)_3$ $(GlcNAc)_2$
3602,8	3602,5	$(Hex)_3$ $(HexNAc)_3$ $(NeuAc)_3$ + $(Man)_3$ $(GlcNAc)_2$
3777,0	3776,6	$(Hex)_3$ $(HexNAc)_3$ $(Dehex)_1$ $(NeuAc)_3$ + $(Man)_3$ $(GlcNAc)_2$

1.2.3. Interpretation

Theoretical mass [M+Na]$^+$	rTSH v22684	Interpretation
1865,9	1866,0	$(Gal)_1$ $(GlcNAc)_2$ + $(Man)_3$ $(GlcNAc)_2$
2070,0	2070,0	$(Gal)_2$ $(GlcNAc)_2$ + $(Man)_3$ $(GlcNAc)_2$
2186,1	2186,1	$(NeuAc)_1$ $(Gal)_1$ $(GlcNAc)_1$ + $(Man)_4$ $(GlcNAc)_2$
2227,1	2227,1	$(NeuAc)_1$ $(Gal)_1$ $(GlcNAc)_2$ + $(Man)_3$ $(GlcNAc)_2$
2431,2	2431,2	$(NeuAc)_1$ $(Gal)_2$ $(GlcNAc)_2$ + $(Man)_3$ $(GlcNAc)_2$
2605,3	2605,2	$(NeuAc)_1$ $(Gal)_2$ $(GlcNAc)_2$ **$(Fuc)_1$** + $(Man)_3$ $(GlcNAc)_2$
2663,3	2663,3	$(Gal)_2$ $(GlcNAc)_3$ **$(Fuc)_2$** + $(Man)_3$ $(GlcNAc)_2$
2792,4	2792,3	$(NeuAc)_2$ $(Gal)_2$ $(GlcNAc)_2$ + $(Man)_3$ $(GlcNAc)_2$
2880,4	2880,3	$(NeuAc)_1$ $(Gal)_3$ $(GlcNAc)_3$ + $(Man)_3$ $(GlcNAc)_2$
2966,5	2966,4	$(NeuAc)_2$ $(Gal)_2$ $(GlcNAc)_2$ **$(Fuc)_1$** + $(Man)_3$ $(GlcNAc)_2$
3054,5	3054,5	$(NeuAc)_1$ $(Gal)_3$ $(GalNAc)_3$ **$(Fuc)_1$** + $(Man)_3$ $(GlcNAc)_2$
3241,6	3241,4	$(NeuAc)_2$ $(Gal)_3$ $(GlcNAc)_3$ + $(Man)_3$ $(GlcNAc)_2$
3415,7	3415,5	$(NeuAc)_2$ $(Gal)_3$ $(GlcNAc)_3$ **$(Fuc)_1$** + $(Man)_3$ $(GlcNAc)_2$
3602,8	3602,5	$(NeuAc)_3$ $(Gal)_3$ $(GlcNAc)_3$ + $(Man)_3$ $(GlcNAc)_2$
3777,0	3776,6	$(NeuAc)_3$ $(Gal)_3$ $(GlcNAc)_3$ **$(Fuc)_1$** + $(Man)_3$ $(GlcNAc)_2$

***Man**: Mannose; **GlcNAc**: N-Acetylglucosamine; **NeuAc**: sialic or N-acetylneuraminic acid; **Fuc**: fucose*

1.2.4. Relative intensity (%)

The following table and figure show the relative intensities of N-glycans present on rec-TSH.

Theoretical mass [M+Na]$^+$	rTSH v22684	Interpretation
1865,9	0,4	$(Gal)_1$ $(GlcNAc)_2$ + $(Man)_3$ $(GlcNAc)_2$
2070,0	5,0	$(Gal)_2$ $(GlcNAc)_2$ + $(Man)_3$ $(GlcNAc)_2$
2186,1	1,2	$(NeuAc)_1$ $(Gal)_1$ $(GlcNAc)_1$ + $(Man)_4$ $(GlcNAc)_2$
2227,1	0,5	$(NeuAc)_1$ $(Gal)_1$ $(GlcNAc)_2$ + $(Man)_3$ $(GlcNAc)_2$
2431,2	36,1	$(NeuAc)_1$ $(Gal)_2$ $(GlcNAc)_2$ + $(Man)_3$ $(GlcNAc)_2$
2605,3	3,4	$(NeuAc)_1$ $(Gal)_2$ $(GlcNAc)_2$ **$(Fuc)_1$** + $(Man)_3$ $(GlcNAc)_2$
2663,3	0,5	$(Gal)_2$ $(GlcNAc)_3$ **$(Fuc)_2$** + $(Man)_3$ $(GlcNAc)_2$
2792,4	31,9	$(NeuAc)_2$ $(Gal)_2$ $(GlcNAc)_2$ + $(Man)_3$ $(GlcNAc)_2$
2880,4	2,7	$(NeuAc)_1$ $(Gal)_3$ $(GlcNAc)_3$ + $(Man)_3$ $(GlcNAc)_2$
2966,5	5,3	$(NeuAc)_2$ $(Gal)_2$ $(GlcNAc)_2$ **$(Fuc)_1$** + $(Man)_3$ $(GlcNAc)_2$
3054,5	0,5	$(NeuAc)_1$ $(Gal)_3$ $(GalNAc)_3$ **$(Fuc)_1$** + $(Man)_3$ $(GlcNAc)_2$
3241,6	6,9	$(NeuAc)_2$ $(Gal)_3$ $(GlcNAc)_3$ + $(Man)_3$ $(GlcNAc)_2$
3415,7	1,5	$(NeuAc)_2$ $(Gal)_3$ $(GlcNAc)_3$ **$(Fuc)_1$** + $(Man)_3$ $(GlcNAc)_2$
3602,8	3,5	$(NeuAc)_3$ $(Gal)_3$ $(GlcNAc)_3$ + $(Man)_3$ $(GlcNAc)_2$
3777,0	0,7	$(NeuAc)_3$ $(Gal)_3$ $(GlcNAc)_3$ **$(Fuc)_1$** + $(Man)_3$ $(GlcNAc)_2$

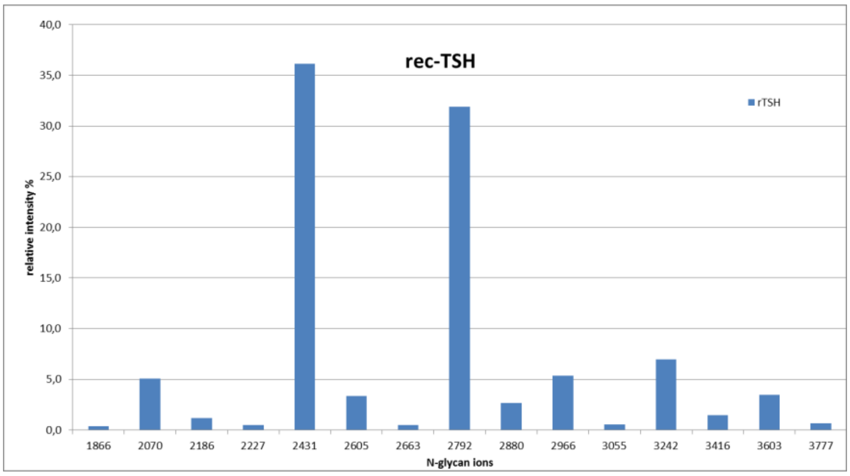

Comparison with previous studies (r TSH: new analysis in green)

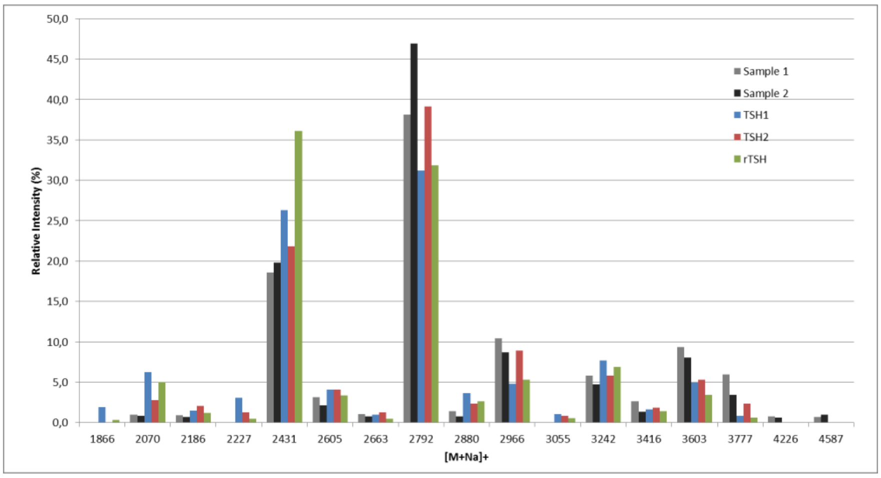

2. Analysis of pituitary hTSH

2.1. Experimental

2.1.1. Glycosidase digestion & permethylation of N-glycans

The pituitary TSH sample pit-TSH (500 µg) was suspended in 200 µl of 50 mM phosphate buffer pH 7.5, denatured in 0.5% sodium dodecyl sulfate (SDS) and 1% β mercaptoethanol (90°C, 10 min) and deglycosylated by enzymatic digestion 15 hours with 20 units of PNGase F (PROMEGA V483A) at 37°C in 50 mM phosphate buffer, pH 7.5 (SOP P27/1).
Deglycosylation was controlled on NuPage gradient 4-12% (Invitrogen), MES buffer, with coomassie staining.

N-glycans were purified on Ultra Clean SPE Carbograph (ALLTECH) according to SOP P24/2. After elution with 25% acetonitrile, 0.1%TFA, the *N*-glycans were lyophilised before permethylation.

A specifically adapted permethylation protocol based on Yu et al., (2009), Glycobiology 19, 1136-1149, using sodium hydroxide, DMSO, ICH3 procedure was used to allow separation of sulphated and non-sulphated or phosphorylated N-glycans. After derivatization, the reaction products were purified on C18 Sep Pak Plus (WATERS) by step-elution with 25% acetonitrile and 50% acetonitrile. The 25% acetonitrile fraction contains sulphated glycans while non-sulphated and phosphorylated glycans are found in the 50% acetonitrile fraction. Eluted N-glycans were lyophilized before MALDI TOF MS.

2.1.2. MALDI TOF analysis of N-glycans

The purified permethylated glycans of the 25% and the 50% acetonitrile elution fractions were solubilised with 20µl of 50:50 acetonitrile/water. N-glycans of each fraction were further purified by binding to ZipTip C18 tip columns. After washing with water glycans of each fraction were eluted in 4 µl of 80% acetonitrile. 1µl of each batch was mixed with 1µl (1 pmoles/µl) of a purified G0F-2AB glycan (m/z 1605) as an internal calibration standard. 2 µl of 2,5 DHB (LaserBiolabs) matrix solution (10mg/ml 50:50 acetonitrile/water) were added and 1 µl aliquots of the glycan+matrix solution were spotted on a MALDI-plate.
Positive ion reflectron MALDI mass spectra were acquired on a VOYAGER DE PRO (AB Sciex).The spectra were obtained by accumulation of 500 shots and were calibrated with an external standard (CalMix 3 AB Sciex). The acceleration and reflector voltage conditions were set up as follows: target voltage 20kV, first grid 75% of target voltage, delayed extraction 150ns.
In this positive acquisition mode non-sulfated permethylated N-glycans are present as classical $[M+Na]^+$ ions. Sulphated N-glycans appear as $[M+(n+1)Na-nH]^+$ ions with n corresponding to the number of sulphate groups present on the glycan.
Interpretation of glycan structures corresponding to monoisotopic masses was performed using EXPAZY GlycoMod tool and GlycoWorkBench for non-sulphated glycans and GycoWorkBench for sulphated N-glycans. PSD fragmentation of selected N-glycans was used to confirm certain assigned structures. MALDI acquisition conditions for PSD were: target voltage 20kV, first grid 80% of target voltage, delayed extraction 120 ns.
Glycan peak heights in the spectra of the 25% and 50% acetonitrile fractions were calibrated using the peak height of the G0F-2AB internal standard within the corresponding spectra.
Normalized peak heights were then used to calculate relative intensities of glycan peaks in each of the two fractions. The overall glycan profile for pit-TSH was generated by compilation of the normalized peak heights of all glycans in the two spectra. Relative intensities (%) were obtained for all N-glycans of pit-TSH by dividing their individual normalized peak heights by the sum of all peak heights in the two spectra.

2.2. Results

2.2.1. MALDI-TOF MS spectra of permethylated N-glycans

25% acetonitrile elution fraction

V22686 cal 22685

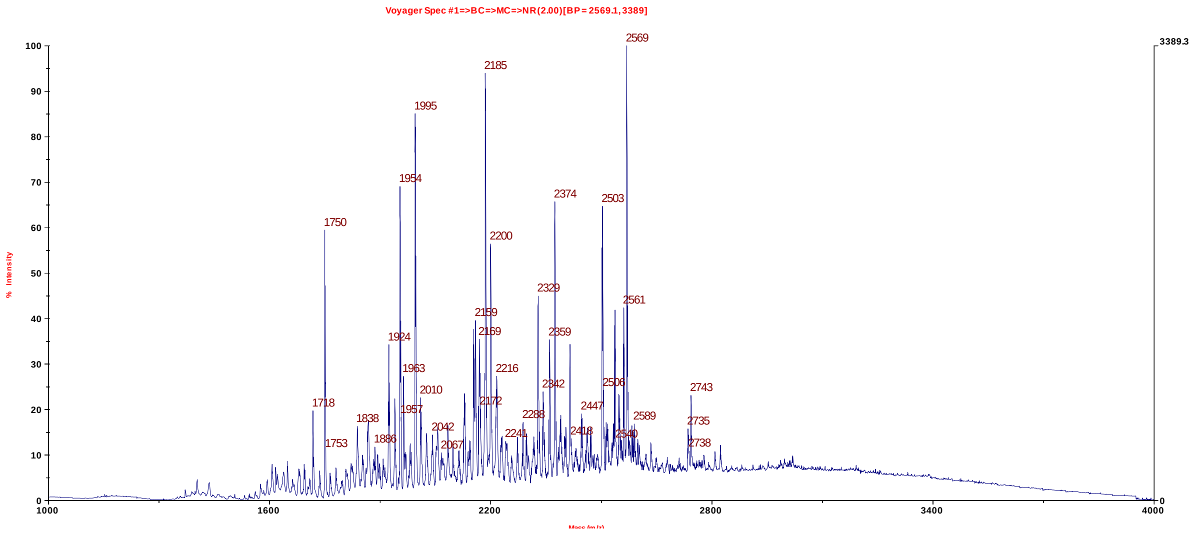

50% acetonitrile elution fraction

V22688 cal 22687

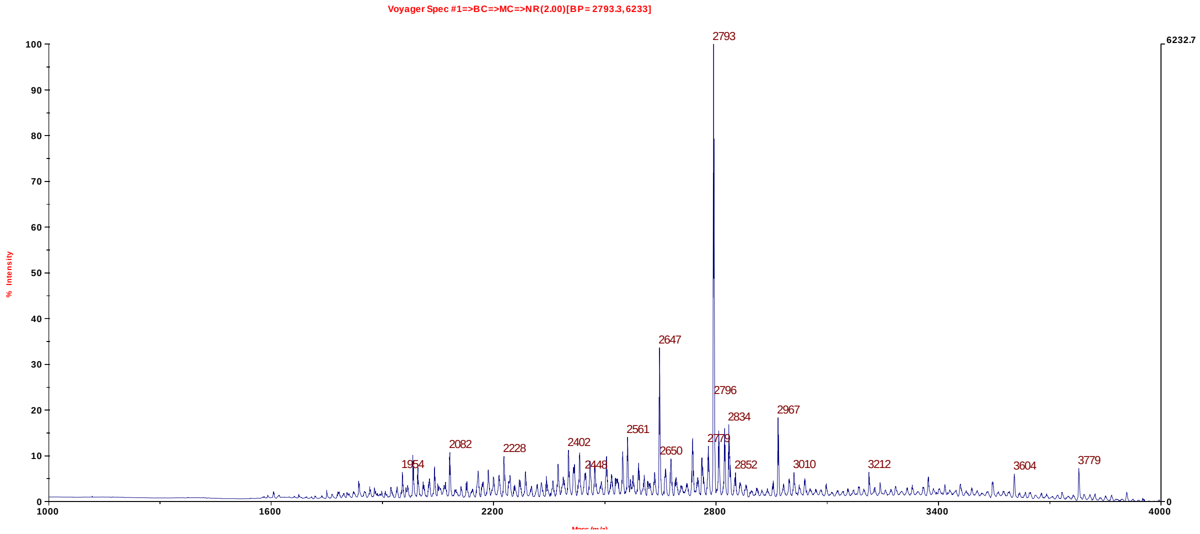

2.2.2. Assignment

Assignment of sulphated N-glycans in the 25% acetonitrile fraction (V22686)

Theoretical Mass	Ion Type	Measured Mass	Assignment
1749,8	[M+2Na-H]$^+$	1749,77	(HexNAc)$_2$ (Sulph)$_1$ + (Man)$_3$ (GlcNAc)$_2$
1923,9	[M+2Na-H]$^+$	1923,84	(HexNAc)$_2$ (Sulph)$_1$ (Dehex)$_1$ + (Man)$_3$ (GlcNAc)$_2$
1953,9	[M+2Na-H]$^+$	1953,86	(Hex)$_1$ (HexNAc)$_2$ (Sulph)$_1$ + (Man)$_3$ (GlcNAc)$_2$
1994,9	[M+2Na-H]$^+$	1994,88	(HexNAc)$_3$ (Sulph)$_1$ + (Man)$_3$ (GlcNAc)$_2$
2128,0	[M+2Na-H]$^+$	2127,94	(Hex)$_1$ (HexNAc)$_2$ (Sulph)$_1$ (Dehex)$_1$ + (Man)$_3$ (GlcNAc)$_2$
2158,0	[M+2Na-H]$^+$	2157,97	(Hex)$_2$ (HexNAc)$_2$ (Sulph)$_1$ + (Man)$_3$ (GlcNAc)$_2$
2169,0	[M+2Na-H]$^+$	2168,97	(HexNAc)$_3$ (Sulph)$_1$ (Dehex)$_1$ + (Man)$_3$ (GlcNAc)$_2$
2185,0	[M+2Na-H]$^+$	2184,98	(NeuAc)$_1$ (Hex)$_1$ (HexNAc)$_3$ (Sulph)$_1$ + (Man)$_3$ (GlcNAc)$_2$, loss of NeuAc*
2199,0	[M+2Na-H]$^+$	2198,98	(Hex)$_{1(}$ HexNAc)$_3$ (Sulph)$_1$ + (Man)$_3$ (GlcNAc)$_2$
2327,9	[M+3Na-2H]$^+$	2327,95	(HexNAc)$_4$ (Sulph)$_2$ + (Man)$_3$ (GlcNAc)$_2$
2359,1	[M+2Na-H]$^+$	2359,06	(NeuAc)$_1$ (Hex)$_1$ (HexNAc)$_3$ (Sulph)$_1$ (Dehex)$_1$ + (Man)$_3$ (GlcNAc)$_2$, loss of NeuAc*
2373,1	[M+2Na-H]$^+$	2373,06	(Hex)$_1$ (HexNAc)$_3$ (Sulph)$_1$ (Dehex)$_1$ + (Man)$_3$ (GlcNAc)$_2$
2414,1	[M+2Na-H]$^+$	2414,11	(HexNAc)$_4$ (Sulph)$_1$ (Dehex)$_1$ + (Man)$_3$ (GlcNAc)$_2$
2502,0	[M+3Na-2H]$^+$	2502,04	(HexNAc)$_4$ (Sulph)$_2$ (Dehex)$_1$ + (Man)$_3$ (GlcNAc)$_2$
2560,2	[M+2Na-H]$^+$	2560,16	(NeuAc)$_1$ (Hex)$_1$ (HexNAc)$_3$ (Sulph)$_1$ + (Man)$_3$ (GlcNAc)$_2$
2568,2	[M-CH$_3$+3Na-H]$^+$	2568,12	(NeuAc)$_1$ (Hex)$_1$ (HexNAc)$_3$ (Sulph)$_1$ + (Man)$_3$ (GlcNAc)$_2$**
2734,3	[M+2Na-H]$^+$	2734,23	(NeuAc)$_1$ (Hex)$_1$ (HexNAc)$_3$ (Sulph)$_1$ (Dehex)$_1$ + (Man)$_3$ (GlcNAc)$_2$
2742,3	[M-CH$_3$+3Na-H]$^+$	2742,22	(NeuAc)$_1$ (Hex)$_1$ (HexNAc)$_3$ (Sulph)$_1$ (Dehex)$_1$ + (Man)$_3$ (GlcNAc)$_2$**

**: ions at m/z 2185 and 2359 are generated by spontaneous fragmentation during MALDI-TOF acquisition of sulphated sialylated parent glycans inducing the loss of the sialic acid (mass loss: 375 Da).*
***: Sulphated sialylated N-glycans can exist in two ion forms, either as [M+2Na –H]$^+$ ions (m/z 2560 and 2734) or as ions that have one less methyl group per sialic acid residue and one more sodium [M- CH$_3$+3Na-H]$^+$.*
The mass difference between the two ion forms is +8 Da. Structures were confirmed by MALDI-PSD fragmentation.

Assignment of N-glycans in the 50% acetonitrile fraction (V22688)

Theoretical Mass [M+Na]$^+$	Measured Mass	Assignment
1835,9	1835,8	(HexNAc)$_2$ (Dehex)$_1$ + (Man)$_3$ (GlcNAc)$_2$
1865,9	1865,8	(Hex)$_1$ (HexNAc)$_2$ + (Man)$_3$ (GlcNAc)$_2$
1982,0	1982,0	(Hex)$_1$ (HexNAc)$_1$ (NeuAc)$_1$ + (Man)$_3$ (GlcNAc)$_2$
2040,0	2040,0	(Hex)$_1$ (HexNAc)$_2$ (Dehex)$_1$ + (Man)$_3$ (GlcNAc)$_2$
2081,1	2081,0	(HexNAc)$_3$ (Dehex)$_1$ + (Man)$_3$ (GlcNAc)$_2$
2156,1	2156,0	(Hex)$_1$ (HexNAc)$_1$ (Dehex)$_1$ (NeuAc)$_1$ + (Man)$_3$ (GlcNAc)$_2$
2227,1	2227,1	(Hex)$_1$ (HexNAc)$_2$ (NeuAc)$_1$ + (Man)$_3$ (GlcNAc)$_2$
2244,1	2244,0	(Hex)$_2$ (HexNAc)$_2$ (Dehex)$_1$ + (Man)$_3$ (GlcNAc)$_2$
2285,2	2285,1	(Hex)$_1$ (HexNAc)$_3$ (Dehex)$_1$ + (Man)$_3$ (GlcNAc)$_2$
2401,2	2401,1	(Hex)$_1$ (HexNAc)$_2$ (Dehex)$_1$ (NeuAc)$_1$ + (Man)$_3$ (GlcNAc)$_2$
2431,2	2431,1	(Hex)$_2$ (HexNAc)$_2$ (NeuAc)$_1$ + (Man)$_3$ (GlcNAc)$_2$
2459,2	2459,1	(Hex)$_1$ (HexNAc)$_3$ (Dehex)$_2$ + (Man)$_3$ (GlcNAc)$_2$
2472,2	2472,1	(Hex)$_1$ (HexNAc)$_3$ (NeuAc)$_1$ + (Man)$_3$ (GlcNAc)$_2$
2560,3	2560,1	(Hex)$_2$ (HexNAc)$_4$ + (Man)$_3$ (GlcNAc)$_2$
2646,2	2646,2	(Hex)$_1$ (HexNAc)$_3$ (Dehex)$_1$ (NeuAc)$_1$ + (Man)$_3$ (GlcNAc)$_2$
2734,4	2734,2	(Hex)$_2$ (HexNAc)$_4$ (Dehex)$_1$ + (Man)$_3$ (GlcNAc)$_2$
2792,4	2792,3	(Hex)$_2$ (HexNAc)$_2$ (NeuAc)$_2$ + (Man)$_3$ (GlcNAc)$_2$
2833,4	2833,3	(Hex)$_1$ (HexNAc)$_3$ (NeuAc)$_2$ + (Man)$_3$ (GlcNAc)$_2$
2966,5	2966,3	(Hex)$_2$ (HexNAc)$_2$ (Dehex)$_1$ (NeuAc)$_2$ + (Man)$_3$ (GlcNAc)$_2$
3211,6	3211,4	(Hex)$_2$ (HexNAc)$_3$ (Dehex)$_1$ (NeuAc)$_2$ + (Man)$_3$ (GlcNAc)
3370,7	3370,4	(Hex)$_3$ (HexNAc)$_5$ (NeuAc)$_1$ + (Man)$_3$ (GlcNAc)$_2$
3544,8	3544,4	(Hex)$_3$ (HexNAc)$_5$ (Dehex)$_1$ (NeuAc)$_1$ + (Man)$_3$ (GlcNAc)$_2$
3602,8	3602,4	(Hex)$_3$ (HexNAc)$_3$ (NeuAc)$_3$ + (Man)$_3$ (GlcNAc)$_2$
3776,9	3776,5	(Hex)$_3$ (HexNAc)$_3$ (Dehex)$_1$ (NeuAc)$_3$ + (Man)$_3$ (GlcNAc)$_2$

2.2.3. Interpretation

Interpretation of sulphated N-glycans in the 25% acetonitrile fraction (V22686)

Theoretical Mass	Ion Type	Measured Mass	Interpretation
1749,8	[M+2Na-H]$^+$	1749,77	(GalNAc)$_1$ (GlcNAc)$_1$ (Sulph)$_1$ + (Man)$_3$ (GlcNAc)$_2$
1923,9	[M+2Na-H]$^+$	1923,84	(GalNAc)$_1$ (GlcNAc)$_1$ (Sulph)$_1$ **(Fuc)$_1$** + (Man)$_3$ (GlcNAc)$_2$
1953,9	[M+2Na-H]$^+$	1953,86	(GalNAc)$_1$ (GlcNAc)$_1$ (Sulph)$_1$ + (Man)$_4$ (GlcNAc)$_2$
1994,9	[M+2Na-H]$^+$	1994,88	(GalNAc)$_1$ (GlcNAc)$_2$ (Sulph)$_1$ + (Man)$_3$ (GlcNAc)$_2$
2128,0	[M+2Na-H]$^+$	2127,94	(GalNAc)$_1$ (GlcNAc)$_1$ (Sulph)$_1$ **(Fuc)$_1$** + (Man)$_4$ (GlcNAc)$_2$
2158,0	[M+2Na-H]$^+$	2157,97	(GalNAc)$_1$ (GlcNAc)$_1$ (Sulph)$_1$ + (Man)$_5$ (GlcNAc)$_2$
2169,0	[M+2Na-H]$^+$	2168,97	(GalNAc)$_1$ (GlcNAc)$_2$ (Sulph)$_1$ **(Fuc)$_1$** + (Man)$_4$ (GlcNAc)$_2$
2185,0	[M+2Na-H]$^+$	2184,98	(NeuAc)$_1$ (Gal)$_1$ (GalNAc)$_1$ (GlcNAc)$_2$ (Sulph)$_1$ + (Man)$_3$ (GlcNAc)$_2$, loss of NeuAc*
2199,0	[M+2Na-H]$^+$	2198,98	(Gal)$_1$ (GalNAc)$_1$ (GlcNAc)$_2$ (Sulph)$_1$ + (Man)$_3$ (GlcNAc)$_2$
2327,9	[M+3Na-2H]$^+$	2327,95	(GalNAc)$_2$ (GlcNAc)$_2$ (Sulph)$_2$ + (Man)$_3$ (GlcNAc)$_2$
2359,1	[M+2Na-H]$^+$	2359,06	(NeuAc)$_1$ (Gal)$_1$ (GalNAc)$_1$ (GlcNAc)$_2$ (Sulph)$_1$ **(Fuc)$_1$** + (Man)$_3$ (GlcNAc)$_2$, loss of NeuAc*
2373,1	[M+2Na-H]$^+$	2373,06	(Gal)$_1$ (GalNAc)$_1$ (GlcNAc)$_2$ (Sulph)$_1$ **(Fuc)$_1$** + (Man)$_3$ (GlcNAc)$_2$
2414,1	[M+2Na-H]$^+$	2414,11	(GalNAc)$_2$ (GlcNAc)$_2$ (Sulph)$_1$ **(Fuc)$_1$** + (Man)$_3$ (GlcNAc)$_2$
2502,0	[M+3Na-2H]$^+$	2502,04	(GalNAc)$_2$ (GlcNAc)$_2$ (Sulph)$_2$ **(Fuc)$_1$** + (Man)$_3$ (GlcNAc)$_2$
2560,2	[M+2Na-H]$^+$	2560,16	(NeuAc)$_1$ (Gal)$_1$ (GalNAc)$_1$ (GlcNAc)$_2$ (Sulph)$_1$ + (Man)$_3$ (GlcNAc)$_2$
2568,2	[M-CH$_3$+3Na-H]$^+$	2568,12	(NeuAc)$_1$ (Gal)$_1$ (GalNAc)$_1$ (GlcNAc)$_2$ (Sulph)$_1$ + (Man)$_3$ (GlcNAc)$_2$**
2734,3	[M+2Na-H]$^+$	2734,23	(NeuAc)$_1$ (Gal)$_1$ (GalNAc)$_1$ (GlcNAc)$_2$ (Sulph)$_1$ **(Fuc)$_1$** + (Man)$_3$ (GlcNAc)$_2$
2742,3	[M-CH$_3$+3Na-H]$^+$	2742,22	(NeuAc)$_1$ (Gal)$_1$ (GalNAc)$_1$ (GlcNAc)$_2$ (Sulph)$_1$ **(Fuc)$_1$** + (Man)$_3$ (GlcNAc)$_2$**

**: ions at m/z 2185 and 2359 are generated by spontaneous fragmentation during MALDI-TOF acquisition of sulphated sialylated parent glycans inducing the loss of the sialic acid (mass loss: 375 Da).*
***: Sulphated sialylated N-glycans can exist in two ion forms, either as [M+2Na –H]$^+$ ions (m/z 2560 and 2734) or as ions that have one less methyl group per sialic acid residue and one more sodium [M-CH$_3$+3Na-H]$^+$. The mass difference between the two ion forms is +8 Da. Structures were confirmed by MALDI-PSD fragmentation.*
***Man**: Mannose; **GlcNAc**: N-Acetylglucosamine; **GalNAc:** N-Acetylgalactosamine; **Sulph**: sulphate; **Fuc**: fucose; **NeuAc**: N-Acetylneuraminic acid*
GalNAc residues are in green, sulphate groups in bright red, NeuAc residues in dark red and fucose is in bold.

Interpretation of N-glycans in the 50% acetonitrile fraction (V22688)

Theoretical Mass [M+Na]$^+$	Measured Mass	Interpretation
1835,9	1835,8	(GlcNAc)$_2$ **(Fuc)$_1$** + (Man)$_3$ (GlcNAc)$_2$
1865,9	1865,8	(Gal)$_1$ (GlcNAc)$_2$ + (Man)$_3$ (GlcNAc)$_2$
1982,0	1982,0	(NeuAc)$_1$ (Gal)$_1$ (GlcNAc)$_1$ + (Man)$_3$ (GlcNAc)$_2$
2040,0	2040,0	(Gal)$_1$ (GlcNAc)$_2$ **(Fuc)$_1$** + (Man)$_3$ (GlcNAc)$_2$
2081,1	2081,0	(GlcNAc)$_3$ **(Fuc)$_1$** + (Man)$_3$ (GlcNAc)$_2$
2156,1	2156,0	(NeuAc)$_1$ (Gal)$_1$ (GlcNAc)$_1$ **(Fuc)$_1$** + (Man)$_3$ (GlcNAc)$_2$
2227,1	2227,1	(NeuAc)$_1$ (Gal)$_1$ (GlcNAc)$_2$ + (Man)$_3$ (GlcNAc)$_2$
2244,1	2244,0	(Gal)$_2$ (GlcNAc)$_2$ **(Fuc)$_1$** + (Man)$_3$ (GlcNAc)$_2$
2285,2	2285,1	(Gal)$_1$ (HexNAc)$_3$ **(Fuc)$_1$** + (Man)$_3$ (GlcNAc)$_2$
2401,2	2401,1	(NeuAc)$_1$ (Gal)$_1$ (GlcNAc)$_2$ **(Fuc)$_1$** + (Man)$_3$ (GlcNAc)$_2$
2431,2	2431,1	(NeuAc)$_1$ (Gal)$_2$ (GlcNAc)$_2$ + (Man)$_3$ (GlcNAc)$_2$
2459,2	2459,1	(Gal)$_1$ (HexNAc)$_3$ **(Fuc)$_2$** + (Man)$_3$ (GlcNAc)$_2$
2472,2	2472,1	(NeuAc)$_1$ (Gal)$_1$ (HexNAc)$_3$ + (Man)$_3$ (GlcNAc)$_2$
2560,3	2560,1	(Gal)$_2$ (HexNAc)$_4$ + (Man)$_3$ (GlcNAc)$_2$
2646,2	2646,2	(NeuAc)$_1$ (Gal)$_1$ (HexNAc)$_3$ **(Fuc)$_1$** + (Man)$_3$ (GlcNAc)$_2$
2734,4	2734,2	(Gal)$_2$ (HexNAc)$_4$ **(Fuc)$_1$** + (Man)$_3$ (GlcNAc)$_2$
2792,4	2792,3	(NeuAc)$_2$ (Gal)$_2$ (GlcNAc)$_2$ + (Man)$_3$ (GlcNAc)$_2$
2833,4	2833,3	(NeuAc)$_2$ (Gal)$_1$ (HexNAc)$_3$ + (Man)$_3$ (GlcNAc)$_2$
2966,5	2966,3	(NeuAc)$_2$ (Gal)$_2$ (GlcNAc)$_2$ **(Fuc)$_1$** + (Man)$_3$ (GlcNAc)$_2$
3211,6	3211,4	(NeuAc)$_2$ (Gal)$_2$ (HexNAc)$_3$ **(Fuc)$_1$** + (Man)$_3$ (GlcNAc)
3370,7	3370,4	(NeuAc)$_1$ (Gal)$_3$ (HexNAc)$_5$ + (Man)$_3$ (GlcNAc)$_2$
3544,8	3544,4	(NeuAc)$_1$ (Gal)$_3$ (HexNAc)$_5$ **(Fuc)$_1$** + (Man)$_3$ (GlcNAc)$_2$
3602,8	3602,4	(NeuAc)$_3$ (Gal)$_3$ (GlcNAc)$_3$ + (Man)$_3$ (GlcNAc)$_2$
3776,9	3776,5	(NeuAc)$_3$ (Gal)$_3$ (GlcNAc)$_3$ **(Fuc)$_1$** + (Man)$_3$ (GlcNAc)$_2$

*For N-glycan structures that may contain different combinations of GlcNAc and GalNAc residues that cannot be distinguished in this analysis the term HexNAc was kept (lines marked in gray). **Man**: Mannose; **GlcNAc**: N-Acetylglucosamine; **NeuAc**: N-acetylneuraminic acid; **Fuc**: fucose*
NeuAc residues are in dark red and fucose is in bold.

2.2.4. Relative intensity (%)

Relative intensity of sulphated N-glycans in the 25% acetonitrile fraction (V22686)

Theoretical Mass	Ion Type	rel. Intensity %	Interpretation
1749,8	[M+2Na-H]$^+$	8,8	(GalNAc)$_1$ (GlcNAc)$_1$ (Sulph)$_1$ + (Man)$_3$ (GlcNAc)$_2$
1923,9	[M+2Na-H]$^+$	2,6	(GalNAc)$_1$ (GlcNAc)$_1$ (Sulph)$_1$ **(Fuc)$_1$** + (Man)$_3$ (GlcNAc)$_2$
1953,9	[M+2Na-H]$^+$	8,9	(GalNAc)$_1$ (GlcNAc)$_1$ (Sulph)$_1$ + (Man)$_4$ (GlcNAc)$_2$
1994,9	[M+2Na-H]$^+$	11,5	(GalNAc)$_1$ (GlcNAc)$_2$ (Sulph)$_1$ + (Man)$_3$ (GlcNAc)$_2$
2128,0	[M+2Na-H]$^+$	1,2	(GalNAc)$_1$ (GlcNAc)$_1$ (Sulph)$_1$ **(Fuc)$_1$** + (Man)$_4$ (GlcNAc)$_2$
2158,0	[M+2Na-H]$^+$	4,3	(GalNAc)$_1$ (GlcNAc)$_1$ (Sulph)$_1$ + (Man)$_5$ (GlcNAc)$_2$
2169,0	[M+2Na-H]$^+$	2,3	(GalNAc)$_1$ (GlcNAc)$_2$ (Sulph)$_1$ **(Fuc)$_1$** + (Man)$_4$ (GlcNAc)$_2$
2185,0	[M+2Na-H]$^+$	10,8	(NeuAc)$_1$ (Gal)$_1$ (GalNAc)$_1$ (GlcNAc)$_2$ (Sulph)$_1$ + (Man)$_3$ (GlcNAc)$_2$, loss of NeuAc*
2199,0	[M+2Na-H]$^+$	6,2	(Gal)$_1$ (GalNAc)$_1$ (GlcNAc)$_2$ (Sulph)$_1$ + (Man)$_3$ (GlcNAc)$_2$
2327,9	[M+3Na-2H]$^+$	5,8	(GalNAc)$_2$ (GlcNAc)$_2$ (Sulph)$_2$ + (Man)$_3$ (GlcNAc)$_2$
2359,1	[M+2Na-H]$^+$	2,8	(NeuAc)$_1$ (Gal)$_1$ (GalNAc)$_1$ (GlcNAc)$_2$ (Sulph)$_1$ **(Fuc)$_1$** + (Man)$_3$ (GlcNAc)$_2$, loss of NeuAc*
2373,1	[M+2Na-H]$^+$	8,0	(Gal)$_1$ (GalNAc)$_1$ (GlcNAc)$_2$ (Sulph)$_1$ **(Fuc)$_1$** + (Man)$_3$ (GlcNAc)$_2$
2414,1	[M+2Na-H]$^+$	2,7	(GalNAc)$_2$ (GlcNAc)$_2$ (Sulph)$_1$ **(Fuc)$_1$** + (Man)$_3$ (GlcNAc)$_2$
2502,0	[M+3Na-2H]$^+$	7,5	(GalNAc)$_2$ (GlcNAc)$_2$ (Sulph)$_2$ **(Fuc)$_1$** + (Man)$_3$ (GlcNAc)$_2$
2560,2	[M+2Na-H]$^+$	4,1	(NeuAc)$_1$ (Gal)$_1$ (GalNAc)$_1$ (GlcNAc)$_2$ (Sulph)$_1$ + (Man)$_3$ (GlcNAc)$_2$
2568,2	[M-CH$_3$+3Na-H]$^+$	9,1	(NeuAc)$_1$ (Gal)$_1$ (GalNAc)$_1$ (GlcNAc)$_2$ (Sulph)$_1$ + (Man)$_3$ (GlcNAc)$_2$**
2734,3	[M+2Na-H]$^+$	1,1	(NeuAc)$_1$ (Gal)$_1$ (GalNAc)$_1$ (GlcNAc)$_2$ (Sulph)$_1$ **(Fuc)$_1$** + (Man)$_3$ (GlcNAc)$_2$
2742,3	[M-CH$_3$+3Na-H]$^+$	2,0	(NeuAc)$_1$ (Gal)$_1$ (GalNAc)$_1$ (GlcNAc)$_2$ (Sulph)$_1$ **(Fuc)$_1$** + (Man)$_3$ (GlcNAc)$_2$**
		100	

Relative intensity of N-glycans in the 50% acetonitrile fraction (V22688)

Theoretical Mass [M+Na]$^+$	relative intensity (%)	Interpretation
1835,9	0,8	(GlcNAc)$_2$ **(Fuc)$_1$** + (Man)$_3$ (GlcNAc)$_2$
1865,9	1,0	(Gal)$_1$ (GlcNAc)$_2$ + (Man)$_3$ (GlcNAc)$_2$
1982,0	4,2	(NeuAc)$_1$ (Gal)$_1$ (GlcNAc)$_1$ + (Man)$_3$ (GlcNAc)$_2$
2040,0	2,3	(Gal)$_1$ (GlcNAc)$_2$ **(Fuc)$_1$** + (Man)$_3$ (GlcNAc)$_2$
2081,1	4,4	(GlcNAc)$_3$ **(Fuc)$_1$** + (Man)$_3$ (GlcNAc)$_2$
2156,1	2,5	(NeuAc)$_1$ (Gal)$_1$ (GlcNAc)$_1$ **(Fuc)$_1$** + (Man)$_3$ (GlcNAc)$_2$
2227,1	3,0	(NeuAc)$_1$ (Gal)$_1$ (GlcNAc)$_2$ + (Man)$_3$ (GlcNAc)$_2$
2244,1	1,5	(Gal)$_2$ (GlcNAc)$_2$ **(Fuc)$_1$** + (Man)$_3$ (GlcNAc)$_2$
2285,2	1,8	(Gal)$_1$ (HexNAc)$_3$ **(Fuc)$_1$** + (Man)$_3$ (GlcNAc)$_2$
2401,2	2,4	(NeuAc)$_1$ (Gal)$_1$ (GlcNAc)$_2$ **(Fuc)$_1$** + (Man)$_3$ (GlcNAc)$_2$
2431,2	2,2	(NeuAc)$_1$ (Gal)$_2$ (GlcNAc)$_2$ + (Man)$_3$ (GlcNAc)$_2$
2459,2	1,9	(Gal)$_1$ (HexNAc)$_3$ **(Fuc)$_2$** + (Man)$_3$ (GlcNAc)$_2$
2472,2	1,8	(NeuAc)$_1$ (Gal)$_1$ (HexNAc)$_3$ + (Man)$_3$ (GlcNAc)$_2$
2560,3	3,2	(Gal)$_2$ (HexNAc)$_4$ + (Man)$_3$ (GlcNAc)$_2$
2646,2	10,2	(NeuAc)$_1$ (Gal)$_1$ (HexNAc)$_3$ **(Fuc)$_1$** + (Man)$_3$ (GlcNAc)$_2$
2734,4	3,3	(Gal)$_2$ (HexNAc)$_4$ **(Fuc)$_1$** + (Man)$_3$ (GlcNAc)$_2$
2792,4	33,7	(NeuAc)$_2$ (Gal)$_2$ (GlcNAc)$_2$ + (Man)$_3$ (GlcNAc)$_2$
2833,4	5,6	(NeuAc)$_2$ (Gal)$_1$ (HexNAc)$_3$ + (Man)$_3$ (GlcNAc)$_2$
2966,5	5,0	(NeuAc)$_2$ (Gal)$_2$ (GlcNAc)$_2$ **(Fuc)$_1$** + (Man)$_3$ (GlcNAc)$_2$
3211,6	1,2	(NeuAc)$_2$ (Gal)$_2$ (HexNAc)$_3$ **(Fuc)$_1$** + (Man)$_3$ (GlcNAc)
3370,7	2,0	(NeuAc)$_1$ (Gal)$_3$ (HexNAc)$_5$ + (Man)$_3$ (GlcNAc)$_2$
3544,8	2,5	(NeuAc)$_1$ (Gal)$_3$ (HexNAc)$_5$ **(Fuc)$_1$** + (Man)$_3$ (GlcNAc)$_2$
3602,8	1,5	(NeuAc)$_3$ (Gal)$_3$ (GlcNAc)$_3$ + (Man)$_3$ (GlcNAc)$_2$
3776,9	2,0	(NeuAc)$_3$ (Gal)$_3$ (GlcNAc)$_3$ **(Fuc)$_1$** + (Man)$_3$ (GlcNAc)$_2$
	100,0	

For N-glycan structures that may contain different combinations of GlcNAc and GalNAc residues that cannot be distinguished in this analysis the term HexNAc was kept (lines marked in gray).

2.2.4. Establishment of a global semi-quantitative profile of N-glycan structures of pit-TSH

The following tables and figure represent the semi-quantitative analysis of all N-glycans present within the 25% and 50% acetonitrile elution fractions of pit-TSH corresponding respectively to spectra V22686 and V22688. N-glycans are sorted according to the glycan ion masses.

The following table represents the normalized peak heights for all N-glycans of the two spectra.

Theoretical Mass	Ion Type	normalized height	Interpretation
1749,8	[M+2Na-H]$^+$	14,8	(GalNAc)$_1$ (GlcNAc)$_1$ (Sulph)$_1$ + (Man)$_3$ (GlcNAc)$_2$
1835,9	[M+Na]$^+$	1,0	(GlcNAc)$_2$ **(Fuc)$_1$** + (Man)$_3$ (GlcNAc)$_2$
1865,9	[M+Na]$^+$	1,2	(Gal)$_1$ (GlcNAc)$_2$ + (Man)$_3$ (GlcNAc)$_2$
1923,9	[M+2Na-H]$^+$	4,4	(GalNAc)$_1$ (GlcNAc)$_1$ (Sulph)$_1$ **(Fuc)$_1$** + (Man)$_3$ (GlcNAc)$_2$
1953,9	[M+2Na-H]$^+$	14,9	(GalNAc)$_1$ (GlcNAc)$_1$ (Sulph)$_1$ + (Man)$_4$ (GlcNAc)$_2$
1982,0	[M+Na]$^+$	5,1	(NeuAc)$_1$ (Gal)$_1$ (GlcNAc)$_1$ + (Man)$_3$ (GlcNAc)$_2$
1994,9	[M+2Na-H]$^+$	19,3	(GalNAc)$_1$ (GlcNAc)$_2$ (Sulph)$_1$ + (Man)$_3$ (GlcNAc)$_2$
2040,0	[M+Na]$^+$	2,8	(Gal)$_1$ (GlcNAc)$_2$ **(Fuc)$_1$** + (Man)$_3$ (GlcNAc)$_2$
2081,1	[M+Na]$^+$	5,2	(GlcNAc)$_3$ **(Fuc)$_1$** + (Man)$_3$ (GlcNAc)$_2$
2128,0	[M+2Na-H]$^+$	2,0	(GalNAc)$_1$ (GlcNAc)$_1$ (Sulph)$_1$ **(Fuc)$_1$** + (Man)$_4$ (GlcNAc)$_2$
2156,1	[M+Na]$^+$	3,0	(NeuAc)$_1$ (Gal)$_1$ (GlcNAc)$_1$ **(Fuc)$_1$** + (Man)$_3$ (GlcNAc)$_2$
2158,0	[M+2Na-H]$^+$	7,2	(GalNAc)$_1$ (GlcNAc)$_1$ (Sulph)$_1$ + (Man)$_5$ (GlcNAc)$_2$
2169,0	[M+2Na-H]$^+$	3,9	(GalNAc)$_1$ (GlcNAc)$_2$ (Sulph)$_1$ **(Fuc)$_1$** + (Man)$_4$ (GlcNAc)$_2$
2185,0	[M+2Na-H]$^+$	18,0	(NeuAc)$_1$ (Gal)$_1$ (GalNAc)$_1$ (GlcNAc)$_2$ (Sulph)$_1$ + (Man)$_3$ (GlcNAc)$_2$, loss of NeuAc*
2199,0	[M+2Na-H]$^+$	10,4	(Gal)$_1$ (GalNAc)$_1$ (GlcNAc)$_2$ (Sulph)$_1$ + (Man)$_3$ (GlcNAc)$_2$
2227,1	[M+Na]$^+$	3,7	(NeuAc)$_1$ (Gal)$_1$ (GlcNAc)$_2$ + (Man)$_3$ (GlcNAc)$_2$
2244,1	[M+Na]$^+$	1,8	(Gal)$_2$ (GlcNAc)$_2$ **(Fuc)$_1$** + (Man)$_3$ (GlcNAc)$_2$
2285,2	[M+Na]$^+$	2,2	(Gal)$_1$ (HexNAc)$_3$ **(Fuc)$_1$** + (Man)$_3$ (GlcNAc)$_2$
2327,9	[M+3Na-2H]$^+$	9,8	(GalNAc)$_2$ (GlcNAc)$_2$ (Sulph)$_2$ + (Man)$_3$ (GlcNAc)$_2$
2359,1	[M+2Na-H]$^+$	4,7	(NeuAc)$_1$ (Gal)$_1$ (GalNAc)$_1$ (GlcNAc)$_2$ (Sulph)$_1$ **(Fuc)$_1$** + (Man)$_3$ (GlcNAc)$_2$, loss of NeuAc*
2373,1	[M+2Na-H]$^+$	13,3	(Gal)$_1$ (GalNAc)$_1$ (GlcNAc)$_2$ (Sulph)$_1$ **(Fuc)$_1$** + (Man)$_3$ (GlcNAc)$_2$
2401,2	[M+Na]$^+$	2,9	(NeuAc)$_1$ (Gal)$_1$ (GlcNAc)$_2$ **(Fuc)$_1$** + (Man)$_3$ (GlcNAc)$_2$
2414,1	[M+2Na-H]$^+$	4,5	(GalNAc)$_2$ (GlcNAc)$_2$ (Sulph)$_1$ **(Fuc)$_1$** + (Man)$_3$ (GlcNAc)$_2$
2431,2	[M+Na]$^+$	2,6	(NeuAc)$_1$ (Gal)$_2$ (GlcNAc)$_2$ + (Man)$_3$ (GlcNAc)$_2$
2459,2	[M+Na]$^+$	2,2	(Gal)$_1$ (HexNAc)$_3$ **(Fuc)$_2$** + (Man)$_3$ (GlcNAc)$_2$
2472,2	[M+Na]$^+$	2,1	(NeuAc)$_1$ (Gal)$_1$ (HexNAc)$_3$ + (Man)$_3$ (GlcNAc)$_2$
2502,0	[M+3Na-2H]$^+$	12,6	(GalNAc)$_2$ (GlcNAc)$_2$ (Sulph)$_2$ **(Fuc)$_1$** + (Man)$_3$ (GlcNAc)$_2$
2560,2	[M+2Na-H]$^+$	6,9	(NeuAc)$_1$ (Gal)$_1$ (GalNAc)$_1$ (GlcNAc)$_2$ (Sulph)$_1$ + (Man)$_3$ (GlcNAc)$_2$
2560,3	[M+Na]$^+$	3,8	(Gal)$_2$ (HexNAc)$_4$ + (Man)$_3$ (GlcNAc)$_2$
2568,2	[M-CH$_3$+3Na-H]$^+$	15,3	(NeuAc)$_1$ (Gal)$_1$ (GalNAc)$_1$ (GlcNAc)$_2$ (Sulph)$_1$ + (Man)$_3$ (GlcNAc)$_2$**
2646,2	[M+Na]$^+$	12,2	(NeuAc)$_1$ (Gal)$_1$ (HexNAc)$_3$ **(Fuc)$_1$** + (Man)$_3$ (GlcNAc)$_2$
2734,3	[M+2Na-H]$^+$	1,9	(NeuAc)$_1$ (Gal)$_1$ (GalNAc)$_1$ (GlcNAc)$_2$ (Sulph)$_1$ **(Fuc)$_1$** + (Man)$_3$ (GlcNAc)$_2$
2734,4	[M+Na]$^+$	4,0	(Gal)$_2$ (HexNAc)$_4$ **(Fuc)$_1$** + (Man)$_3$ (GlcNAc)$_2$
2742,3	[M-CH$_3$+3Na-H]$^+$	3,3	(NeuAc)$_1$ (Gal)$_1$ (GalNAc)$_1$ (GlcNAc)$_2$ (Sulph)$_1$ **(Fuc)$_1$** + (Man)$_3$ (GlcNAc)$_2$**
2792,4	[M+Na]$^+$	40,5	(NeuAc)$_2$ (Gal)$_2$ (GlcNAc)$_2$ + (Man)$_3$ (GlcNAc)$_2$
2833,4	[M+Na]$^+$	6,7	(NeuAc)$_2$ (Gal)$_1$ (HexNAc)$_3$ + (Man)$_3$ (GlcNAc)$_2$
2966,5	[M+Na]$^+$	6,0	(NeuAc)$_2$ (Gal)$_2$ (GlcNAc)$_2$ **(Fuc)$_1$** + (Man)$_3$ (GlcNAc)$_2$
3211,6	[M+Na]$^+$	1,4	(NeuAc)$_2$ (Gal)$_2$ (HexNAc)$_3$ **(Fuc)$_1$** + (Man)$_3$ (GlcNAc)
3370,7	[M+Na]$^+$	2,4	(NeuAc)$_1$ (Gal)$_3$ (HexNAc)$_5$ + (Man)$_3$ (GlcNAc)$_2$
3544,8	[M+Na]$^+$	3,0	(NeuAc)$_1$ (Gal)$_3$ (HexNAc)$_5$ **(Fuc)$_1$** + (Man)$_3$ (GlcNAc)$_2$
3602,8	[M+Na]$^+$	1,8	(NeuAc)$_3$ (Gal)$_3$ (GlcNAc)$_3$ + (Man)$_3$ (GlcNAc)$_2$
3776,9	[M+Na]$^+$	2,4	(NeuAc)$_3$ (Gal)$_3$ (GlcNAc)$_3$ **(Fuc)$_1$** + (Man)$_3$ (GlcNAc)$_2$

For N-glycan structures that may contain different combinations of GlcNAc and GalNAc residues that cannot be distinguished in this analysis the term HexNAc was kept (lines marked in gray).

Relative intensities are calculated in the following table:

Theoretical Mass	Ion Type	relative intensity %	Interpretation
1749,8	[M+2Na-H]$^+$	5,1	(GalNAc)$_1$ (GlcNAc)$_1$ (Sulph)$_1$ + (Man)$_3$ (GlcNAc)$_2$
1835,9	[M+Na]$^+$	0,3	(GlcNAc)$_2$ **(Fuc)$_1$** + (Man)$_3$ (GlcNAc)$_2$
1865,9	[M+Na]$^+$	0,4	(Gal)$_1$ (GlcNAc)$_2$ + (Man)$_3$ (GlcNAc)$_2$
1923,9	[M+2Na-H]$^+$	1,5	(GalNAc)$_1$ (GlcNAc)$_1$ (Sulph)$_1$ **(Fuc)$_1$** + (Man)$_3$ (GlcNAc)$_2$
1953,9	[M+2Na-H]$^+$	5,2	(GalNac)$_1$ (GlcNAc)$_1$ (Sulph)$_1$ + (Man)$_4$ (GlcNAc)$_2$
1982,0	[M+Na]$^+$	1,8	(NeuAc)$_1$ (Gal)$_1$ (GlcNAc)$_1$ + (Man)$_3$ (GlcNAc)$_2$
1994,9	[M+2Na-H]$^+$	6,7	(GalNAc)$_1$ (GlcNAc)$_2$ (Sulph)$_1$ + (Man)$_3$ (GlcNAc)$_2$
2040,0	[M+Na]$^+$	1,0	(Gal)$_1$ (GlcNAc)$_2$ **(Fuc)$_1$** + (Man)$_3$ (GlcNAc)$_2$
2081,1	[M+Na]$^+$	1,8	(GlcNAc)$_3$ **(Fuc)$_1$** + (Man)$_3$ (GlcNAc)$_2$
2128,0	[M+2Na-H]$^+$	0,7	(GalNAc)$_1$ (GlcNAc)$_1$ (Sulph)$_1$ **(Fuc)$_1$** + (Man)$_4$ (GlcNAc)$_2$
2156,1	[M+Na]$^+$	1,1	(NeuAc)$_1$ (Gal)$_1$ (GlcNAc)$_1$ **(Fuc)$_1$** + (Man)$_3$ (GlcNAc)$_2$
2158,0	[M+2Na-H]$^+$	2,5	(GalNac)$_1$ (GlcNAc)$_1$ (Sulph)$_1$ + (Man)$_5$ (GlcNAc)$_2$
2169,0	[M+2Na-H]$^+$	1,4	(GalNAc)$_1$ (GlcNAc)$_2$ (Sulph)$_1$ **(Fuc)$_1$** + (Man)$_4$ (GlcNAc)$_2$
2185,0	[M+2Na-H]$^+$	6,3	(NeuAc)$_1$ (Gal)$_1$ (GalNAc)$_1$ (GlcNAc)$_2$ (Sulph)$_1$ + (Man)$_3$ (GlcNAc)$_2$, loss of NeuAc*
2199,0	[M+2Na-H]$^+$	3,6	(Gal)$_1$ (GalNAc)$_1$ (GlcNAc)$_2$ (Sulph)$_1$ + (Man)$_3$ (GlcNAc)$_2$
2227,1	[M+Na]$^+$	1,3	(NeuAc)$_1$ (Gal)$_1$ (GlcNAc)$_2$ + (Man)$_3$ (GlcNAc)$_2$
2244,1	[M+Na]$^+$	0,6	(Gal)$_2$ (GlcNAc)$_2$ **(Fuc)$_1$** + (Man)$_3$ (GlcNAc)$_2$
2285,2	[M+Na]$^+$	0,8	(Gal)$_1$ (HexNAc)$_3$ **(Fuc)$_1$** + (Man)$_3$ (GlcNAc)$_2$
2327,9	[M+3Na-2H]$^+$	3,4	(GalNAc)$_2$ (GlcNAc)$_2$ (Sulph)$_2$ + (Man)$_3$ (GlcNAc)$_2$
2359,1	[M+2Na-H]$^+$	1,7	(NeuAc)$_1$ (Gal)$_1$ (GalNAc)$_1$ (GlcNAc)$_2$ (Sulph)$_1$ **(Fuc)$_1$** + (Man)$_3$ (GlcNAc)$_2$, loss of NeuAc*
2373,1	[M+2Na-H]$^+$	4,6	(Gal)$_1$ (GalNAc)$_1$ (GlcNAc)$_2$ (Sulph)$_1$ **(Fuc)$_1$** + (Man)$_3$ (GlcNAc)$_2$
2401,2	[M+Na]$^+$	1,0	(NeuAc)$_1$ (Gal)$_1$ (GlcNAc)$_2$ **(Fuc)$_1$** + (Man)$_3$ (GlcNAc)$_2$
2414,1	[M+2Na-H]$^+$	1,6	(GalNAc)$_2$ (GlcNAc)$_2$ (Sulph)$_1$ **(Fuc)$_1$** + (Man)$_3$ (GlcNAc)$_2$
2431,2	[M+Na]$^+$	0,9	(NeuAc)$_1$ (Gal)$_2$ (GlcNAc)$_2$ + (Man)$_3$ (GlcNAc)$_2$
2459,2	[M+Na]$^+$	0,8	(Gal)$_1$ (HexNAc)$_3$ **(Fuc)$_2$** + (Man)$_3$ (GlcNAc)$_2$
2472,2	[M+Na]$^+$	0,7	(NeuAc)$_1$ (Gal)$_1$ (HexNAc)$_3$ + (Man)$_3$ (GlcNAc)$_2$
2502,0	[M+3Na-2H]$^+$	4,4	(GalNAc)$_2$ (GlcNAc)$_2$ (Sulph)$_2$ **(Fuc)$_1$** + (Man)$_3$ (GlcNAc)$_2$
2560,2	[M+2Na-H]$^+$	2,4	(NeuAc)$_1$ (Gal)$_1$ (GalNAc)$_1$ (GlcNAc)$_2$ (Sulph)$_1$ + (Man)$_3$ (GlcNAc)$_2$
2560,3	[M+Na]$^+$	1,3	(Gal)$_2$ (HexNAc)$_4$ + (Man)$_3$ (GlcNAc)$_2$
2568,2	[M-CH$_3$+3Na-H]$^+$	5,3	(NeuAc)$_1$ (Gal)$_1$ (GalNAc)$_1$ (GlcNAc)$_2$ (Sulph)$_1$ + (Man)$_3$ (GlcNAc)$_2$**
2646,2	[M+Na]$^+$	4,2	(NeuAc)$_1$ (Gal)$_1$ (HexNAc)$_3$ **(Fuc)$_1$** + (Man)$_3$ (GlcNAc)$_2$
2734,3	[M+2Na-H]$^+$	0,6	(NeuAc)$_1$ (Gal)$_1$ (GalNAc)$_1$ (GlcNAc)$_2$ (Sulph)$_1$ **(Fuc)$_1$** + (Man)$_3$ (GlcNAc)$_2$
2734,4	[M+Na]$^+$	1,4	(Gal)$_2$ (HexNAc)$_4$ **(Fuc)$_1$** + (Man)$_3$ (GlcNAc)$_2$
2742,3	[M-CH$_3$+3Na-H]$^+$	1,2	(NeuAc)$_1$ (Gal)$_1$ (GalNAc)$_1$ (GlcNAc)$_2$ (Sulph)$_1$ **(Fuc)$_1$** + (Man)$_3$ (GlcNAc)$_2$**
2792,4	[M+Na]$^+$	14,1	(NeuAc)$_2$ (Gal)$_2$ (GlcNAc)$_2$ + (Man)$_3$ (GlcNAc)$_2$
2833,4	[M+Na]$^+$	2,3	(NeuAc)$_2$ (Gal)$_1$ (HexNAc)$_3$ + (Man)$_3$ (GlcNAc)$_2$
2966,5	[M+Na]$^+$	2,1	(NeuAc)$_2$ (Gal)$_2$ (GlcNAc)$_2$ **(Fuc)$_1$** + (Man)$_3$ (GlcNAc)$_2$
3211,6	[M+Na]$^+$	0,5	(NeuAc)$_2$ (Gal)$_2$ (HexNAc)$_3$ **(Fuc)$_1$** + (Man)$_3$ (GlcNAc)
3370,7	[M+Na]$^+$	0,8	(NeuAc)$_1$ (Gal)$_3$ (HexNAc)$_5$ + (Man)$_3$ (GlcNAc)$_2$
3544,8	[M+Na]$^+$	1,0	(NeuAc)$_1$ (Gal)$_3$ (HexNAc)$_5$ **(Fuc)$_1$** + (Man)$_3$ (GlcNAc)$_2$
3602,8	[M+Na]$^+$	0,6	(NeuAc)$_3$ (Gal)$_3$ (GlcNAc)$_3$ + (Man)$_3$ (GlcNAc)$_2$
3776,9	[M+Na]$^+$	0,9	(NeuAc)$_3$ (Gal)$_3$ (GlcNAc)$_3$ **(Fuc)$_1$** + (Man)$_3$ (GlcNAc)$_2$
		100,0	

For N-glycan structures that may contain different combinations of GlcNAc and GalNAc residues that cannot be distinguished in this analysis the term HexNAc was kept (lines marked in gray).

The table contains ions at m/z 2185, 2560 and 2568 that correspond all to the sialylated sulphated glycan (NeuAc)$_1$ (Gal)$_1$ (GalNAc)$_1$ (GlcNAc)$_2$ (Sulph)$_1$ + (Man)$_3$ (GlcNAc)$_2$. In addition ions m/z 2359, 2734 and 2742 correspond all to the glycan (NeuAc)$_1$ (Gal)$_1$ (GalNAc)$_1$ (GlcNAc)$_2$ (Sulph)$_1$ **(Fuc)$_1$**+ (Man)$_3$ (GlcNAc)$_2$. In the following table the sum of relative intensities of the different ions for each of these two N-glycans is listed for the corresponding [M+2Na-H]$^+$ ions to determine the overall percentage of the corresponding sulphated N-glycan within the glycan population of pit-TSH

Theoretical Mass	Ion Type	relative intensity %	Interpretation
1749,8	[M+2Na-H]$^+$	5,1	(GalNAc)$_1$ (GlcNAc)$_1$ (Sulph)$_1$ + (Man)$_3$ (GlcNAc)$_2$
1835,9	[M+Na]$^+$	0,3	(GlcNAc)$_2$ **(Fuc)$_1$** + (Man)$_3$ (GlcNAc)$_2$
1865,9	[M+Na]$^+$	0,4	(Gal)$_1$ (GlcNAc)$_2$ + (Man)$_3$ (GlcNAc)$_2$
1923,9	[M+2Na-H]$^+$	1,5	(GalNAc)$_1$ (GlcNAc)$_1$ (Sulph)$_1$ **(Fuc)$_1$** + (Man)$_3$ (GlcNAc)$_2$
1953,9	[M+2Na-H]$^+$	5,2	(GalNAc)$_1$ (GlcNAc)$_1$ (Sulph)$_1$ + (Man)$_4$ (GlcNAc)$_2$
1982,0	[M+Na]$^+$	1,8	(NeuAc)$_1$ (Gal)$_1$ (GlcNAc)$_1$ + (Man)$_3$ (GlcNAc)$_2$
1994,9	[M+2Na-H]$^+$	6,7	(GalNAc)$_1$ (GlcNAc)$_2$ (Sulph)$_1$ + (Man)$_3$ (GlcNAc)$_2$
2040,0	[M+Na]$^+$	1,0	(Gal)$_1$ (GlcNAc)$_2$ **(Fuc)$_1$** + (Man)$_3$ (GlcNAc)$_2$
2081,1	[M+Na]$^+$	1,8	(GlcNAc)$_3$ **(Fuc)$_1$** + (Man)$_3$ (GlcNAc)$_2$
2128,0	[M+2Na-H]$^+$	0,7	(GalNAc)$_1$ (GlcNAc)$_1$ (Sulph)$_1$ **(Fuc)$_1$** + (Man)$_4$ (GlcNAc)$_2$
2156,1	[M+Na]$^+$	1,1	(NeuAc)$_1$ (Gal)$_1$ (GlcNAc)$_1$ **(Fuc)$_1$** + (Man)$_3$ (GlcNAc)$_2$
2158,0	[M+2Na-H]$^+$	2,5	(GalNAc)$_1$ (GlcNAc)$_1$ (Sulph)$_1$ + (Man)$_5$ (GlcNAc)$_2$
2169,0	[M+2Na-H]$^+$	1,4	(GalNAc)$_1$ (GlcNAc)$_2$ (Sulph)$_1$ **(Fuc)$_1$** + (Man)$_4$ (GlcNAc)$_2$
2199,0	[M+2Na-H]$^+$	3,6	(Gal)$_1$ (GalNAc)$_1$ (GlcNAc)$_2$ (Sulph)$_1$ + (Man)$_3$ (GlcNAc)$_2$
2227,1	[M+Na]$^+$	1,3	(NeuAc)$_1$ (Gal)$_1$ (GlcNAc)$_2$ + (Man)$_3$ (GlcNAc)$_2$
2244,1	[M+Na]$^+$	0,6	(Gal)$_2$ (GlcNAc)$_2$ **(Fuc)$_1$** + (Man)$_3$ (GlcNAc)$_2$
2285,2	[M+Na]$^+$	0,8	(Gal)$_1$ (HexNAc)$_3$ **(Fuc)$_1$** + (Man)$_3$ (GlcNAc)$_2$
2327,9	[M+3Na-2H]$^+$	3,4	(GalNAc)$_2$ (GlcNAc)$_2$ (Sulph)$_2$ + (Man)$_3$ (GlcNAc)$_2$
2373,1	[M+2Na-H]$^+$	4,6	(Gal)$_1$ (GalNAc)$_1$ (GlcNAc)$_2$ (Sulph)$_1$ **(Fuc)$_1$** + (Man)$_3$ (GlcNAc)$_2$
2401,2	[M+Na]$^+$	1,0	(NeuAc)$_1$ (Gal)$_1$ (GlcNAc)$_2$ **(Fuc)$_1$** + (Man)$_3$ (GlcNAc)$_2$
2414,1	[M+2Na-H]$^+$	1,6	(GalNAc)$_2$ (GlcNAc)$_2$ (Sulph)$_1$ **(Fuc)$_1$** + (Man)$_3$ (GlcNAc)$_2$
2431,2	[M+Na]$^+$	0,9	(NeuAc)$_1$ (Gal)$_2$ (GlcNAc)$_2$ + (Man)$_3$ (GlcNAc)$_2$
2459,2	[M+Na]$^+$	0,8	(Gal)$_1$ (HexNAc)$_3$ **(Fuc)$_2$** + (Man)$_3$ (GlcNAc)$_2$
2472,2	[M+Na]$^+$	0,7	(NeuAc)$_1$ (Gal)$_1$ (HexNAc)$_3$ + (Man)$_3$ (GlcNAc)$_2$
2502,0	[M+3Na-2H]$^+$	4,4	(GalNAc)$_2$ (GlcNAc)$_2$ (Sulph)$_2$ **(Fuc)$_1$** + (Man)$_3$ (GlcNAc)$_2$
**2560,2**	[M+2Na-H]$^+$	14,0	(NeuAc)$_1$ (Gal)$_1$ (GalNAc)$_1$ (GlcNAc)$_2$ (Sulph)$_1$ + (Man)$_3$ (GlcNAc)$_2$
2560,3	[M+Na]$^+$	1,3	(Gal)$_2$ (HexNAc)$_4$ + (Man)$_3$ (GlcNAc)$_2$
2646,2	[M+Na]$^+$	4,2	(NeuAc)$_1$ (Gal)$_1$ (HexNAc)$_3$ **(Fuc)$_1$** + (Man)$_3$ (GlcNAc)$_2$
**2734,3**	[M+2Na-H]$^+$	3,5	(NeuAc)$_1$ (Gal)$_1$ (GalNAc)$_1$ (GlcNAc)$_2$ (Sulph)$_1$ **(Fuc)$_1$** + (Man)$_3$ (GlcNAc)$_2$
2734,4	[M+Na]$^+$	1,4	(Gal)$_2$ (HexNAc)$_4$ **(Fuc)$_1$** + (Man)$_3$ (GlcNAc)$_2$
2792,4	[M+Na]$^+$	14,1	(NeuAc)$_2$ (Gal)$_2$ (GlcNAc)$_2$ + (Man)$_3$ (GlcNAc)$_2$
2833,4	[M+Na]$^+$	2,3	(NeuAc)$_2$ (Gal)$_1$ (HexNAc)$_3$ + (Man)$_3$ (GlcNAc)$_2$
2966,5	[M+Na]$^+$	2,1	(NeuAc)$_2$ (Gal)$_2$ (GlcNAc)$_2$ **(Fuc)$_1$** + (Man)$_3$ (GlcNAc)$_2$
3211,6	[M+Na]$^+$	0,5	(NeuAc)$_2$ (Gal)$_2$ (HexNAc)$_3$ **(Fuc)$_1$** + (Man)$_3$ (GlcNAc)
3370,7	[M+Na]$^+$	0,8	(NeuAc)$_1$ (Gal)$_3$ (HexNAc)$_5$ + (Man)$_3$ (GlcNAc)$_2$
3544,8	[M+Na]$^+$	1,0	(NeuAc)$_1$ (Gal)$_3$ (HexNAc)$_5$ **(Fuc)$_1$** + (Man)$_3$ (GlcNAc)$_2$
3602,8	[M+Na]$^+$	0,6	(NeuAc)$_3$ (Gal)$_3$ (GlcNAc)$_3$ + (Man)$_3$ (GlcNAc)$_2$
3776,9	[M+Na]$^+$	0,9	(NeuAc)$_3$ (Gal)$_3$ (GlcNAc)$_3$ **(Fuc)$_1$** + (Man)$_3$ (GlcNAc)$_2$
		100,0	

For N-glycan ions in bold the indicated percentage corresponds to the sum of the relative intensities of the different forms of the corresponding glycans that are present in the spectra.
For N-glycan structures that may contain different combinations of GlcNAc and GalNAc residues that cannot be distinguished in this analysis the term HexNAc was kept (lines marked in gray).

The following figure represents the overall semi-quantitative N-glycan profile of native pituitary hTSH.

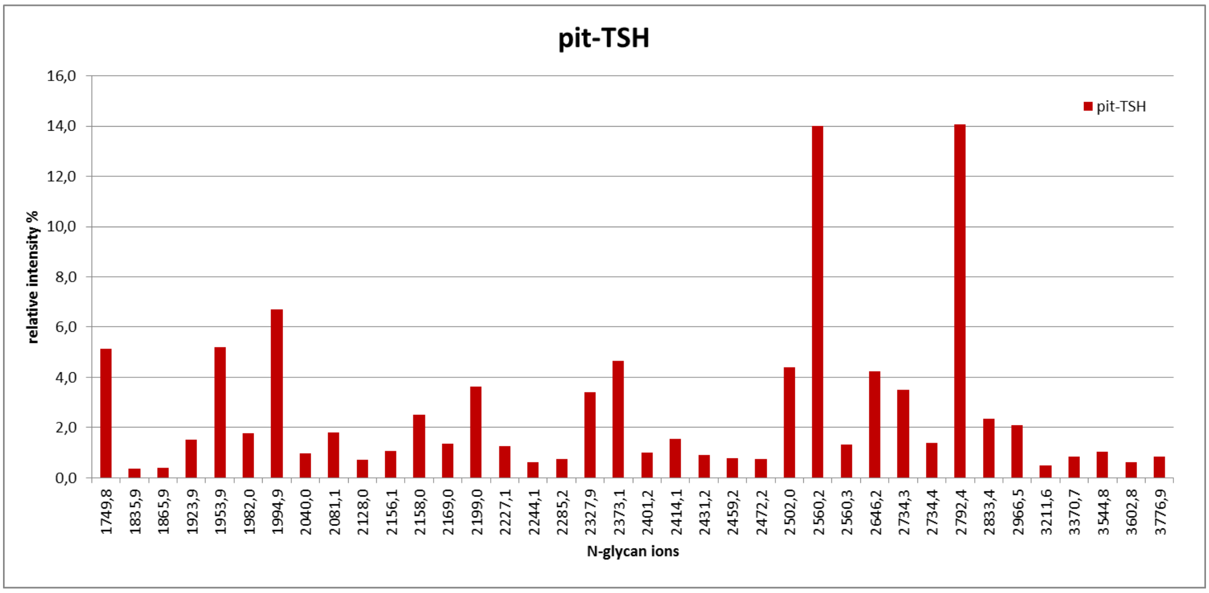

3. Conclusions

N-glycan profiles of recombinant hTSH from CHO cells and native pituitary hTSH show significant differences concerning glycan composition. The native molecule contains sulphated N-glycan structures that are not present on the recombinant molecules.
The following table and figure show a comparison of the N-glycan profiles of the two molecules.

Theoretical Mass	relativ intensity	relativ intensity	Interpretation
	pit-TSH	rec-TSH	
1749,8	5,1	0,0	$(GalNAc)_1$ $(GlcNAc)_1$ $(Sulph)_1$ + $(Man)_3$ $(GlcNAc)_2$
1835,9	0,3	0,0	$(GlcNAc)_2$ **$(Fuc)_1$** + $(Man)_3$ $(GlcNAc)_2$
1865,9	0,4	0,4	$(Gal)_1$ $(GlcNAc)_2$ + $(Man)_3$ $(GlcNAc)_2$
1923,9	1,5	0,0	$(GalNAc)_1$ $(GlcNAc)_1$ $(Sulph)_1$ **$(Fuc)_1$** + $(Man)_3$ $(GlcNAc)_2$
1953,9	5,2	0,0	$(GalNac)_1$ $(GlcNAc)_1$ $(Sulph)_1$ + $(Man)_4$ $(GlcNAc)_2$
1982,0	1,8	0,0	$(NeuAc)_1$ $(Gal)_1$ $(GlcNAc)_1$ + $(Man)_3$ $(GlcNAc)_2$
1994,9	6,7	0,0	$(GalNAc)_1$ $(GlcNAc)_2$ $(Sulph)_1$ + $(Man)_3$ $(GlcNAc)_2$
2040,0	1,0	0,0	$(Gal)_2$ $(GlcNAc)_2$ + $(Man)_3$ $(GlcNAc)_2$
2070,0	0	5,0	$(Gal)_1$ $(GlcNAc)_2$ **$(Fuc)_1$** + $(Man)_3$ $(GlcNAc)_2$
2081,1	1,8	0,0	$(GlcNAc)_3$ **$(Fuc)_1$** + $(Man)_3$ $(GlcNAc)_2$
2128,0	0,7	0,0	$(GalNAc)_1$ $(GlcNAc)_1$ $(Sulph)_1$ **$(Fuc)_1$** + $(Man)_4$ $(GlcNAc)_2$
2156,1	1,1	0,0	$(NeuAc)_1$ $(Gal)_1$ $(GlcNAc)_1$ **$(Fuc)_1$** + $(Man)_3$ $(GlcNAc)_2$
2158,0	2,5	0,0	$(GalNac)_1$ $(GlcNAc)_1$ $(Sulph)_1$ + $(Man)_5$ $(GlcNAc)_2$
2169,0	1,4	0,0	$(GalNAc)_1$ $(GlcNAc)_2$ $(Sulph)_1$ **$(Fuc)_1$** + $(Man)_4$ $(GlcNAc)_2$
2186,1	0	1,2	$(NeuAc)_1$ $(Gal)_1$ $(GlcNAc)_1$ + $(Man)_4$ $(GlcNAc)_2$
2199,0	3,6	0,0	$(Gal)_1$ $(GalNAc)_1$ $(GlcNAc)_2$ $(Sulph)_1$ + $(Man)_3$ $(GlcNAc)_2$
2227,1	1,3	0,5	$(NeuAc)_1$ $(Gal)_1$ $(GlcNAc)_2$ + $(Man)_3$ $(GlcNAc)_2$
2244,1	0,6	0,0	$(Gal)_2$ $(GlcNAc)_2$ **$(Fuc)_1$** + $(Man)_3$ $(GlcNAc)_2$
2285,2	0,8	0,0	$(Gal)_1$ $(HexNAc)_3$ **$(Fuc)_1$** + $(Man)_3$ $(GlcNAc)_2$
2327,9	3,4	0,0	$(GalNAc)_2$ $(GlcNAc)_2$ $(Sulph)_2$ + $(Man)_3$ $(GlcNAc)_2$
2373,1	4,6	0,0	$(Gal)_1$ $(GalNAc)_1$ $(GlcNAc)_2$ $(Sulph)_1$ **$(Fuc)_1$** + $(Man)_3$ $(GlcNAc)_2$
2401,2	1,0	0,0	$(NeuAc)_1$ $(Gal)_1$ $(GlcNAc)_2$ **$(Fuc)_1$** + $(Man)_3$ $(GlcNAc)_2$
2414,1	1,6	0,0	$(GalNAc)_2$ $(GlcNAc)_2$ $(Sulph)_1$ **$(Fuc)_1$** + $(Man)_3$ $(GlcNAc)_2$
2431,2	0,9	36,1	$(NeuAc)_1$ $(Gal)_2$ $(GlcNAc)_2$ + $(Man)_3$ $(GlcNAc)_2$
2459,2	0,8	0,0	$(Gal)_1$ $(HexNAc)_3$ **$(Fuc)_2$** + $(Man)_3$ $(GlcNAc)_2$
2472,2	0,7	0,0	$(NeuAc)_1$ $(Gal)_1$ $(HexNAc)_3$ + $(Man)_3$ $(GlcNAc)_2$
2502,0	4,4	0,0	$(GalNAc)_2$ $(GlcNAc)_2$ $(Sulph)_2$ **$(Fuc)_1$** + $(Man)_3$ $(GlcNAc)_2$
2560,2	14,0	0,0	$(NeuAc)_1$ $(Gal)_1$ $(GalNAc)_1$ $(GlcNAc)_2$ $(Sulph)_1$ + $(Man)_3$ $(GlcNAc)_2$
2560,3	1,3	0,0	$(Gal)_2$ $(HexNAc)_4$ + $(Man)_3$ $(GlcNAc)_2$
2605,3	0	3,4	$(NeuAc)_1$ $(Gal)_2$ $(GlcNAc)_2$ **$(Fuc)_1$** + $(Man)_3$ $(GlcNAc)_2$
2646,2	4,2	0,0	$(NeuAc)_1$ $(Gal)_1$ $(HexNAc)_3$ **$(Fuc)_1$** + $(Man)_3$ $(GlcNAc)_2$
2663,3	0	0,5	$(Gal)_2$ $(GlcNAc)_3$ **$(Fuc)_2$** + $(Man)_3$ $(GlcNAc)_2$
2734,3	3,5	0,0	$(NeuAc)_1$ $(Gal)_1$ $(GalNAc)_1$ $(GlcNAc)_2$ $(Sulph)_1$ **$(Fuc)_1$** + $(Man)_3$ $(GlcNAc)_2$
2734,4	1,4	0,0	$(Gal)_2$ $(HexNAc)_4$ **$(Fuc)_1$** + $(Man)_3$ $(GlcNAc)_2$
2792,4	14,1	31,9	$(NeuAc)_2$ $(Gal)_2$ $(GlcNAc)_2$ + $(Man)_3(GlcNAc)_2$
2833,4	2,3	0,0	$(NeuAc)_2$ $(Gal)_1$ $(HexNAc)_3$ + $(Man)_3(GlcNAc)_2$
2880,4	0	2,7	$(NeuAc)_1$ $(Gal)_3$ $(GclNAc)_3$ + $(Man)_3(GlcNAc)_2$
2966,5	2,1	5,3	$(NeuAc)_2$ $(Gal)_2$ $(GlcNAc)_2$ **$(Fuc)_1$** + $(Man)_3(GlcNAc)_2$
3054,5	0	0,5	$(NeuAc)_1$ $(Gal)_3$ $(GlcNAc)_3$ **$(Fuc)_1$** + $(Man)_3(GlcNAc)_2$
3211,6	0,5	0,0	$(NeuAc)_2$ $(Gal)_2$ $(HexNAc)_3$ **$(Fuc)_1$** + $(Man)_3(GlcNAc)$
3241,6	0	6,9	$(NeuAc)_2$ $(Gal)_3$ $(GlcNAc)_3$ + $(Man)_3(GlcNAc)_2$
3370,7	0,8	0,0	$(NeuAc)_1$ $(Gal)_3$ $(HexNAc)_5$ + $(Man)_3(GlcNAc)_2$
3415,7	0	1,5	$(NeuAc)_2$ $(Gal)_3$ $(GlcNAc)_3$ **$(Fuc)_1$** + $(Man)_3(GlcNAc)_2$
3544,8	1,0	0,0	$(NeuAc)_1$ $(Gal)_3$ $(HexNAc)_5$ **$(Fuc)_1$** + $(Man)_3(GlcNAc)_2$
3602,8	0,6	3,5	$(NeuAc)_3$ $(Gal)_3$ $(GlcNAc)_3$ + $(Man)_3(GlcNAc)_2$
3776,9	0,9	0,7	$(NeuAc)_3$ $(Gal)_3$ $(GlcNAc)_3$ **$(Fuc)_1$** + $(Man)_3(GlcNAc)_2$

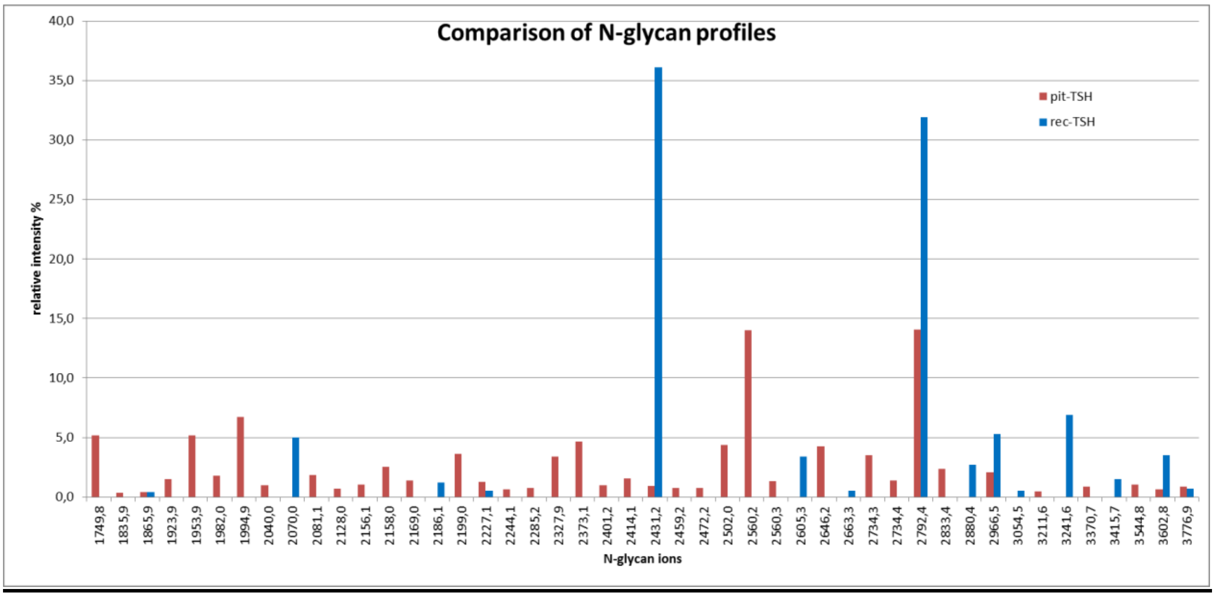
Comparison of N-glycan profiles
pit-TSH
rec-TSH
relative intensity %
40,0
35,0
30,0
25,0
20,0
15,0
10,0
5,0
0,0
N-glycan ions
1749,8
1835,9
1865,9
1923,9
1953,9
1982,0
1994,9
2040,0
2070,0
2081,1
2128,0
2156,1
2158,0
2169,0
2186,1
2199,0
2227,1
2244,1
2285,2
2327,9
2373,1
2401,2
2414,1
2431,2
2459,2
2472,2
2502,0
2560,2
2560,3
2605,3
2646,2
2663,3
2734,3
2734,4
2792,4
2833,4
2880,4
2966,5
3054,5
3211,6
3241,6
3370,7
3415,7
3544,8
3602,8
3776,9
